# Development of solvent-free synthesis of hydrogen-bonded supramolecular polyurethanes†
†Electronic supplementary information (ESI) available: Synthetic procedures, characterisation, and additional data associated with optimisation and substrate tolerance studies. See DOI: 10.1039/c4sc03804e



**DOI:** 10.1039/c4sc03804e

**Published:** 2015-02-03

**Authors:** Kelly. A. Houton, George M. Burslem, Andrew. J. Wilson

**Affiliations:** a School of Chemistry , University of Leeds , Woodhouse Lane , Leeds LS2 9JT , UK . Email: A.J.Wilson@leeds.ac.uk ; Fax: +44 (0)113 3436565 ; Tel: +44 (0)113 3431409; b Astbury Centre for Structural Molecular Biology , University of Leeds , Woodhouse Lane , Leeds LS2 9JT , UK

## Abstract

In this work we describe the solvent free synthesis of supramolecular polyurethanes held together by heterocomplementary triple hydrogen bonding. We perform a systematic evaluation on the base catalyzed synthesis of small molecule ureas and carbamates from a range of isocyanates, amines and alcohols in solution and in the solid state using ball milling. These optimized procedures are then shown to be applicable to the synthesis of supramolecular polyurethanes using solvent-free methods.

## Abstract 2

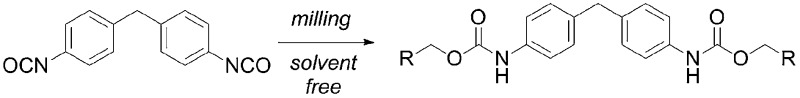
A solvent free ball-milling method for the synthesis of small molecule and oligomeric carbamates is described that is applicable to supramolecular polymer synthesis.

## Introduction

A major goal in synthetic (polymer) chemistry is the development of synthetic methods that limit environmental impact through elimination of (harmful) solvents, use of lower temperatures and accelerated reaction time *e.g.* through catalysis.^1^ In terms of solvent, the use of supercritical CO_2_ has proven useful but is limited to suitably soluble polymers.^2^ Similarly, ionic liquids may be used,^3^ although extraction of the product polymer still needs consideration. Mechanochemical organic synthesis, currently undergoing a revival,^4–6^ has been used to promote Knoevenagel condensations,^7^ Aldol reactions^8^ and Michael additions^9^ amongst many other synthetically important organic transformations.^10^ Significantly, mechanochemical synthesis has also been applied to the synthesis of organic frameworks^11,12^ and crystalline materials.^12,13^ Supramolecular polymers have generated enormous interest during the last 10–15 years^14,15^ as a consequence of the opportunities to study assembly mechanisms^16–19^ at a fundamental level, and, due to the stimuli-responsive properties they possess, application in a wide array of settings.^20,21^ Moreover, supramolecular polymers offer opportunities for the design of synthetic methods, given that synthesis of the low molecular weight building blocks may be more amenable to common strategies employed to limit environmental impact. Although covalent mechanochemical syntheses have been extensively studied,^5^ we found no literature precedent for supramolecular polymer material synthesis, although note the use of ultrasound (which has found use in covalent synthesis)^22^ to assemble/disassemble co-ordination polymers.^23^

**Fig. 1:**
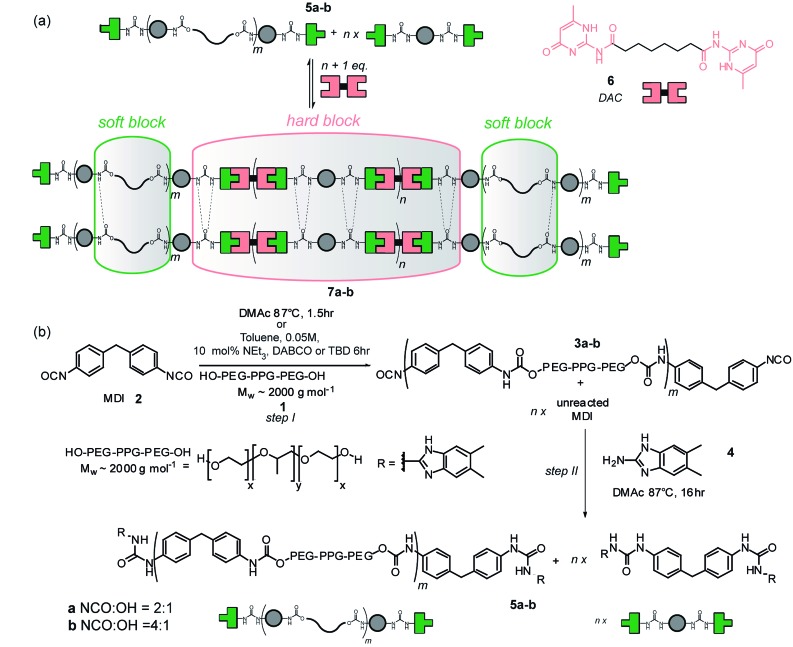
Supramolecular polyurethane (PU) synthesis and assembly (a) schematic depicting assembly of a supramolecular PU elastomer mediated by triple hydrogen-bonding between amido*iso*cytosine and ureidoimidazole (b) synthesis of the supramolecular macromolecular components; reaction of telechelic diol with MDI and then amine leading to the ureidoimidazole recognition motif provides a statistical mixture of chain elongated telechelic (denoted m) and capped MDI (denoted *n*). Addition of *n* + 1 equivalents of ditopic diamidoisocytosine **6** results in matched stoichiometry of the hydrogen-bonding units.

Our group previously developed a series of hydrogen bonding motifs,^24–28^ with which to construct supramolecular polymers^29–31^ and employed a ureidoimidazole/amido*iso*cytosine triply hydrogen bonded diad^26^ for assembly of supramolecular polyurethanes^31–33^ (Fig. 1a). These were prepared *via* the ‘prepolymer’ approach (Fig. 1b), where an amorphous diol **1** is reacted with MDI **2** giving an isocyanate terminated pre-polymer **3** which is then end capped with hydrogen bonding units to form a macromonomer **5**. Upon addition of a supramolecular chain extender **6**, phase separation occurs, conferring elastomeric properties upon the material **7**. The amorphous diol allows deformation of the material whilst the hydrogen bonding units contained within the crystalline phase promote retention of the original material configuration.^34^ Synthesis of the macromonomer using this method is a two-step one-pot reaction requiring the temperature to be maintained at 87 °C for 17.5 h (Fig. 1b).^31^ Furthermore, the dimethylacetamide (DMAc) solvent is high boiling, hygroscopic and teratogenic. Polyurethanes have established themselves in many areas of our everyday life, and their production is subject to constant growth.^35,36^ Solvent-free syntheses are desirable due to the difficulty in obtaining a solvent, which is suitable for solubilizing high percentage hard block containing phase separated polymers;^37^ polymers with high urea content are liable to gelation and premature precipitation, so often require high temperatures and highly polar solvents. Our supramolecular polyurethane elastomer synthesis therefore represented an ideal model to develop milder reaction conditions and explore a solvent free synthetic route. Herein we describe such a study by first outlining a systematic study on urea and carbamate formation in both the solution and the solid state and then the application of our findings to the synthesis of a supramolecular polyurethane.

## Results and discussion

The primary approach used to synthesize polyurethanes (PUs) is the polyaddition of a diisocyanate and a telechelic diol, usually in the presence of a catalyst, which generally fall into two categories; organotins or amines^38^ (although acids have also recently been employed).^39^ This simple reaction combined with a wide range of commercially available isocyanate and alcohol starting materials results in an extensive array of products.^38^ However, the reaction is not necessarily clean; four degradation pathways have been identified, as well as self-cyclization to form dimers and trimers.^38^ As temperature increases, increased byproduct formation occurs; reversion to starting material, olefin formation and urethane rearrangement *via* transcarbamoylation all occur due to the weak C–NH bond.^38^ The high nucleophilicity of water towards isocyanates can also result in the formation of a urea with the release of CO_2_ (foaming reaction), hence synthesis of PUs normally requires anhydrous conditions.^40^ PU catalysis is well known in the literature,^36,38,41,42^ Organometallic dibutyltin dilaurate is extensively used on an industrial scale,^43^ but residual amounts of the compound affect the lifetime of the product^44^ and the environmental impact of using heavy metals is substantial. Similarly, hindered organic bases have been shown to catalyze urethane formation *e.g.* diazabicyclo[2.2.2]octane (DABCO), although this too exhibits toxicity and its reduced usage is desirable.^36,38^ Finally, the melt *trans*-urethane reaction has been used for PU synthesis although higher reaction temperatures are required.^45^

### Optimization of solution phase synthesis

**Scheme 1:**
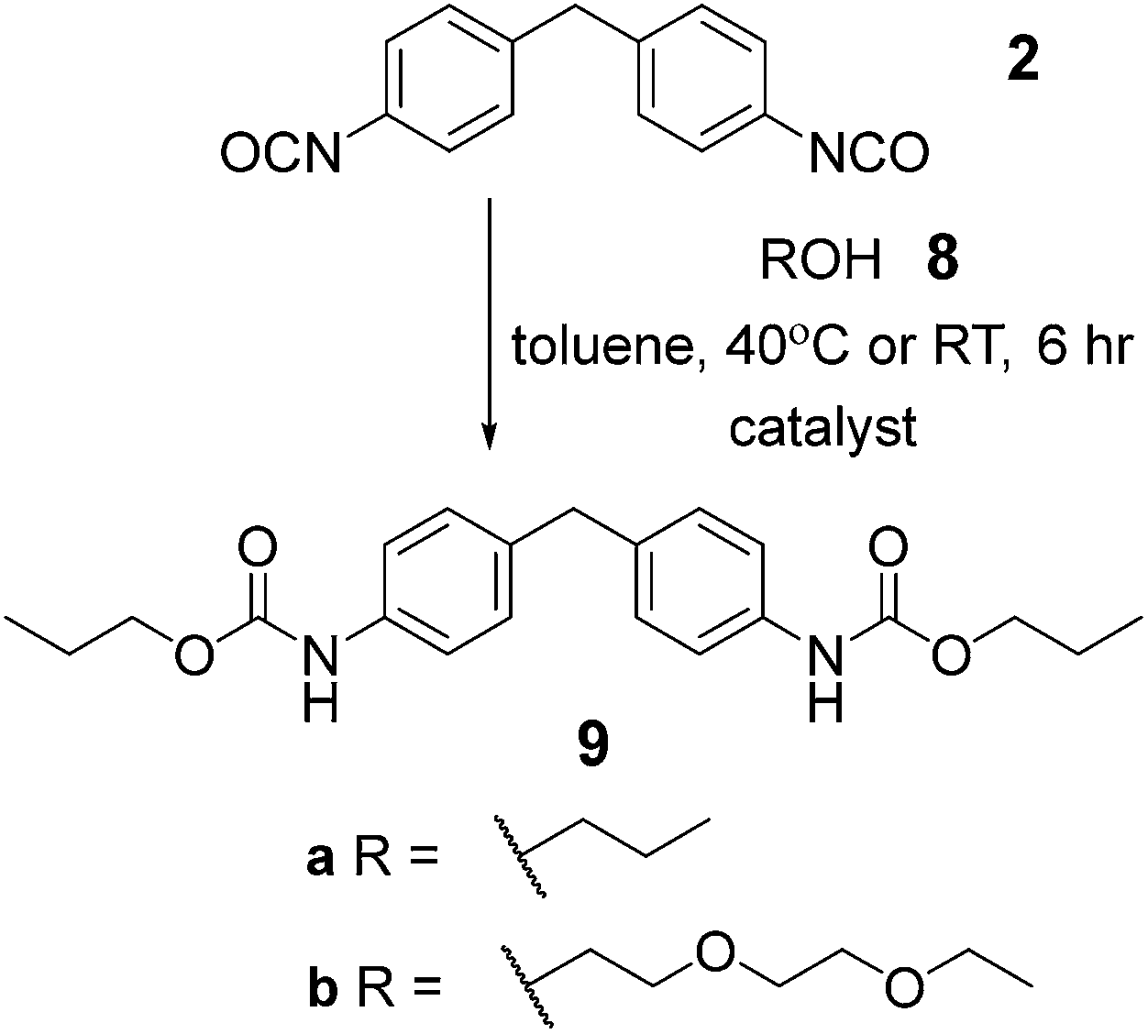
Synthetic conditions applied to model reactions.

To have a reference point for our studies on solid-state synthesis of urethanes, it was necessary to develop optimized solution phase conditions. Burkus previously reported conditions for triethylamine (Et_3_N) catalyzed carbamate formation.^46^ We applied these and several variants on the catalyst to a model reaction (Scheme 1, Table 1 and Fig. S1–7†) between 1-propanol **8a** and 4,4-methylene-bis-(phenylisocyanate) (MDI) **2**. Using triethylamine as catalyst, precipitation of the carbamate **9a** was evident after 1 hour and afforded product in over 60% yield. These conditions replace the use of DMAc with toluene and reduce the temperature required for carbamate formation relative to the conditions used for pre-polymer synthesis, which is likely to reduce side reactions.^42^ Subsequent studies on the effect of catalyst, catalyst p*K*_a_ and reaction temperature (Fig. S1–7†) revealed DABCO and Et_3_N to be superior catalysts whereas 1,5,7-triazabicyclo[4.4.0]dec-5-ene (TBD) was found to retard conversion to biscarbamate. In general, a moderate increase in temperature did not improve the conversion under all conditions (Fig. S2 and 3†). In each case the reactions produced carbamate, the urea side product of the foaming reaction (Fig. S8†) and unreacted starting material. Consistent with the literature for DABCO,^47^ reduced foaming and improved yield was evident for DABCO and NEt_3_, whereas TBD produced additional side products (also consistent with the literature)^41^ (Fig. S1–3†). A clear correlation between catalyst p*K*_a_ and carbamate formation was observed; as p*K*_a_ increases (a property proposed to promote foaming),^48^ conversion decreases (Table 1). Time dependence of the reaction was studied qualitatively by IR, observing the loss of isocyanate absorption at 2200 cm^–1^ with time together with appearance of an absorption at 1739 cm^–1^ for the carbamate C=O (Fig. S9†); the rate at which starting material is consumed appears to increase with time which is consistent with autocatalysis of the reaction.^49^

**Table 1:** Effect of catalyst on conversion of **2** to **9a–b** ^*a*^

Entry	Catalyst	p*K*_a_	Temp/°C	Conversion to **9a** ^*b*^	Conversion to **9b** ^*b*^
1	None	—	RT	29	84
2	None	—	40	29	88
3	Et_3_N	10.8	RT	75	84
4	Et_3_N	10.8	40	74	89
5	DABCO	8.9	RT	91	88
6	DABCO	8.9	40	97	92
7	TBD	22	RT	67	63
8	TBD	22	40	66	62

^*a*^Conditions: reaction concentration 0.05 M, 10 mol% catalyst (entries 3–8) used, 1 equiv. of **2**, 2 equiv. of propanol **8a** or **8b** diethylene glycol monoethyl ether, toluene, 6 h.

^*b*^Conversion to **9** is based on comparison of NMR signals of crude reaction mixture.

To complete our optimization study, we performed a solvent screen and probed the effect of reaction concentration using Et_3_N as catalyst (see Fig. S10 and S11† for details). At higher concentrations, gelation occurred after 10 minutes indicating formation of a cross-linked network, (potentially through lateral hydrogen bonding of carbamate groups)^50^ preventing further conversion of starting materials. The lowest concentration gave the best result (100% conversion to product at 0.01 M). From the solvent screen, toluene was identified as most suitable; this might indicate a role for catalysis of urethane formation *via* hydrogen bond activated complexes.^51^ Finally, we repeated a subset of experiments using diethyleneglycol-mono-ethyl ether **8b** (see Fig. S12–21†) as a small molecule mimic of the PEG–PPG–PEG telechelic to be used in supramolecular polymer synthesis; similar results were obtained to those with propanol **8a** (Table 1).

These conditions were then applied to the synthesis of a polyurethane macromonomer **3a** (NCO : OH ratio 2 : 1, Fig. 1b, step I) with IR used to monitor reaction progress (Fig. S22†). The ^1^H NMR resonances in this polymeric system, affected our ability to differentiate between urea and urethane NH and aromatic protons, hence we could only determine conversion from isocyanate. Here, catalyst effect on conversion was less pronounced (although for TBD with the higher p*K*_a_, the reaction was less clean), whilst temperature did not affect overall conversion (Fig. S23 and 24†). For entries 5, 7 and 8 a product that was insoluble in DMSO-*d*_6_ was obtained; gelation during these reactions and subsequent insolubility could suggest a *pseudo* Tromsdorff (gel) effect, which is common in polymerization procedures (Table2).^52^

**Table 2:** Effect of catalyst on conversion of **2** to **3a** ^*a*^

Entry	Catalyst	p*K*_a_	Temp/°C	Reaction conversion^*b*^
1	None	—	RT	90
2	None	—	40	95
3	NEt_3_	10.8	RT	88
4	NEt_3_	10.8	40	87
5	DABCO	8.9	RT	Not soluble
6	DABCO	8.9	40	100
7	TBD	22	RT	Not soluble
8	TBD	22	40	Not soluble

^*a*^Conditions: reaction concentration 0.05 M, 10 mol% catalyst (entries 3–8) used, 2 equiv. of **2**, 1 equiv. of polyol **1**, toluene, 6 h.

^*b*^Reaction of NCO is based on comparison of NMR signals of crude reaction mixture.

### Development of solvent free syntheses

With an efficient catalyst identified and several parameters optimized in solution phase, we investigated solvent-free synthesis using mechanochemistry. We found limited evidence for urea/carbamate synthesis^53^ using ball milling but note it has been shown to effect acylation reactions, thioisocyante and thiourea synthesis.^54,55^ MDI **2** and 1-propanol **8a** and diethyleneglycol-mono-ethyl ether **8b** were used as model substrates to explore whether carbamate **9a–b** formation was possible using mechanochemical methods. For this study, the ball mill was set to a vibrational frequency of 20 Hz for three minute intervals and the reaction monitored by IR. All three catalysts were again tested for activity in the bulk state and compared to the uncatalyzed reaction under the same reaction conditions (Fig. S25–34†).

**Fig. 2:**
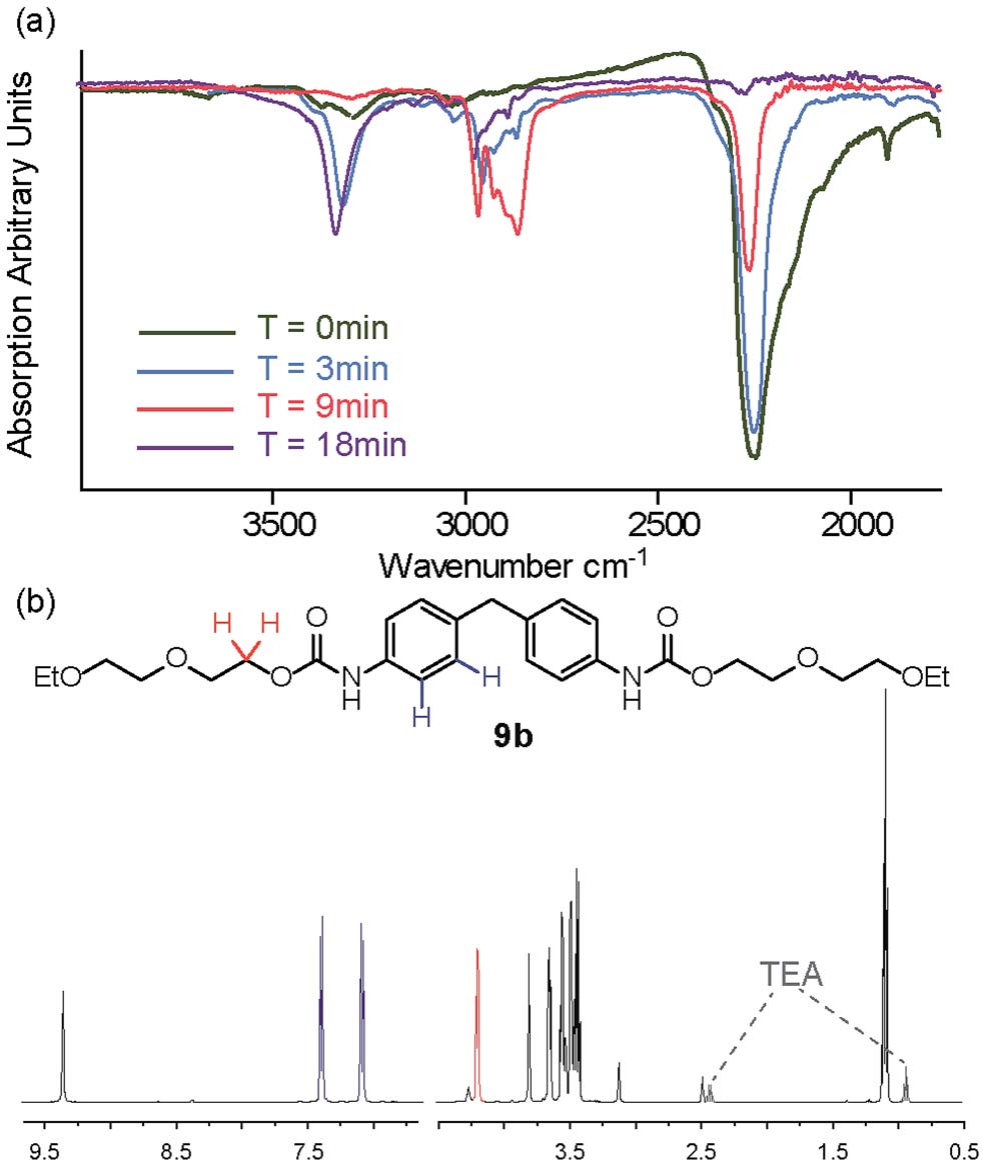
Solvent free carbamate syntheses (a) IR spectra showing the effect of ball milling at three minute intervals on carbamate **9a** formation; a decrease in absorption at 2200 cm^–1^ indicates consumption of starting material. (b) ^1^H NMR (500 MHz, DMSO-*d*_6_) of the crude product **9b** obtained from reaction of **2** and **8b**.

For 1-propanol **8a** after three minutes, a decrease in isocyanate absorbance at 2200 cm^–1^ was indicative of consumption of starting material and the reaction beginning to take place (Fig. 2a). A change in frequency and sharpening of the stretch at ∼3300 cm^–1^ was also observed consistent with loss of alcohol (OH stretch) and formation of a carbamate (NH stretch). After eighteen minutes of milling, there was no IR absorption for the isocyanate or –OH stretch for three of the reactions, highlighting similar reactivity for the Et_3_N, DABCO and uncatalysed reactions (entries 1–3, Table 3) and indicating a less pronounced role for the catalyst although TBD was again detrimental to carbamate **9a** formation. Similarly synthesis of carbamate **9b** occurred with good conversion as evidenced in the crude NMR spectra, a resonance at 4.23 ppm expected for the OC*H*_2_–CO–NH group being diagnostic (Fig. 2b).

**Table 3:** Effect of catalyst on conversion of **2** to **9a–b** ^*a*^

Entry	Catalyst	p*K*_a_	Conversion to **9a** ^*b*^	Conversion to **9b** ^*b*^
1	None	—	93	86
2	Et_3_N	10.8	95	98
3	DABCO	8.9	93	98
4	TBD	22	80	87

^*a*^Conditions: 10 mol% catalyst (entries 2–4) used, 1 equiv. of **2**, 2 equiv. of propanol **8**, vibrational frequency was 20 Hz, 18 min.

^*b*^Conversion to **9** is based on comparison of NMR signals of crude reaction mixture.

**Scheme 2:**
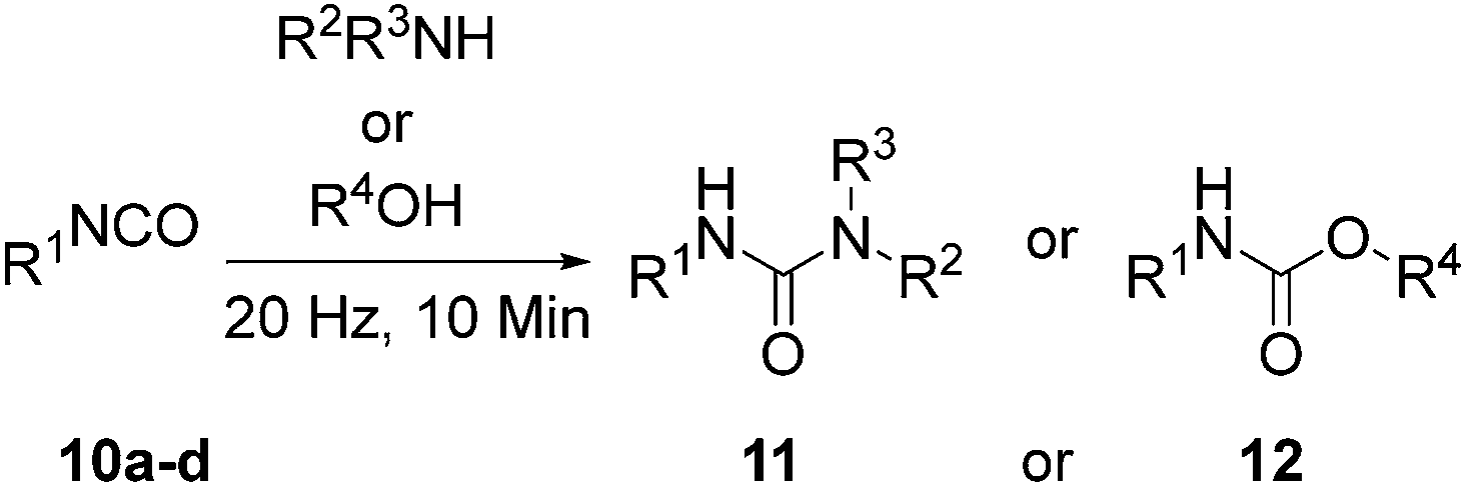
Solvent free carbamate/urea synthesis.

Having shown milling to effectively promote the model reaction without a catalyst, substrate tolerance to the reaction conditions was assessed for urea **11** and carbamate **12** formation so as to ascertain the effectiveness of the method for small molecule synthesis. An isocyanate and either an amine or alcohol (Scheme 2) were tested under these conditions (Table 4).

**Table 4:** Substrate tolerance for solvent free carbamate/urea synthesis^*a*^

Conversion^*b*^ (%)	R^2^ or R^4^	R^3^	R^1^	Product
46^*c*^	Benzimidazole	H	*p*-CN benzene	**11a**
99	CH(C_2_H_6_)	CH(C_2_H_6_)	*p*-NO_2_ benzene	**11b**
100	CH(C_2_H_6_)	CH(C_2_H_6_)	*p*-C_2_H_5_ benzene	**11c**
60^*c*^	Benzimidazole	H	–adamantane	**11d**
100	CH(C_2_H_6_)	CH(C_2_H_6_)	–adamantane	**11e**
79	C_3_H_5_		*p*-NO_2_ benzene	**12a**
100	C_3_H_5_		–adamantane	**12b**
100^*c*^	CH_2_ *O*-nitrobenzene		–adamantane	**12c**
50^*c*^	CH_2_ *O*-nitrobenzene		*p*-CN benzene	**12d**
75^*c*^	CH_2_ *O*-nitrobenzene		*p*-NO_2_ benzene	**12e**
55^*c*^	CH_2_ *O*-bromobenzene		*p*-C_2_H_5_ benzene	**12f**

^*a*^Conditions: no catalyst, 1 equiv. of isocyanate **10**, 1 equiv. of amine/alcohol (2 equiv. in the case of MDI), vibrational frequency was 20 Hz, 10 min.

^*b*^Conversion to **11**/**12** is based on comparison of NMR signals of crude reaction mixture.

^*c*^Donates a solid–solid reaction.

In our hands – the method is tolerant to a broad array of functionality (Fig. S35–45†). Isocyanates bearing electron withdrawing substituents **11a–b** generally increased reactivity whereas ethyl substituent **11c** reacts less well presumably due to the inductive effect of the alkyl group.^56^ Poorer nucleophiles work less well. Many of the ureas synthesized using this method are heavily hindered and we were unable to obtain some of these *via* traditional solution methodology. Solid amines, isocyanates and alcohols in addition to liquid counterparts were tested. All substrates were found to be amenable to these conditions indicating that liquid assisted grinding (LAG) effects^57^ are negligible in promoting the reaction. This is important given that both the model reaction (MDI and 1-propanol) and the supramolecular macromonomer synthesis (see below) are both reactions that involve a liquid component. It is also noteworthy that in a recently described acid catalyzed carbamate synthesis which was performed under ball milling conditions, the reaction took place with a liquid catalyst,^39^ hence the current results unequivocally illustrate the utility of catalyst free, ball milling for solvent free urea and carbamate synthesis.

**Scheme 3:**
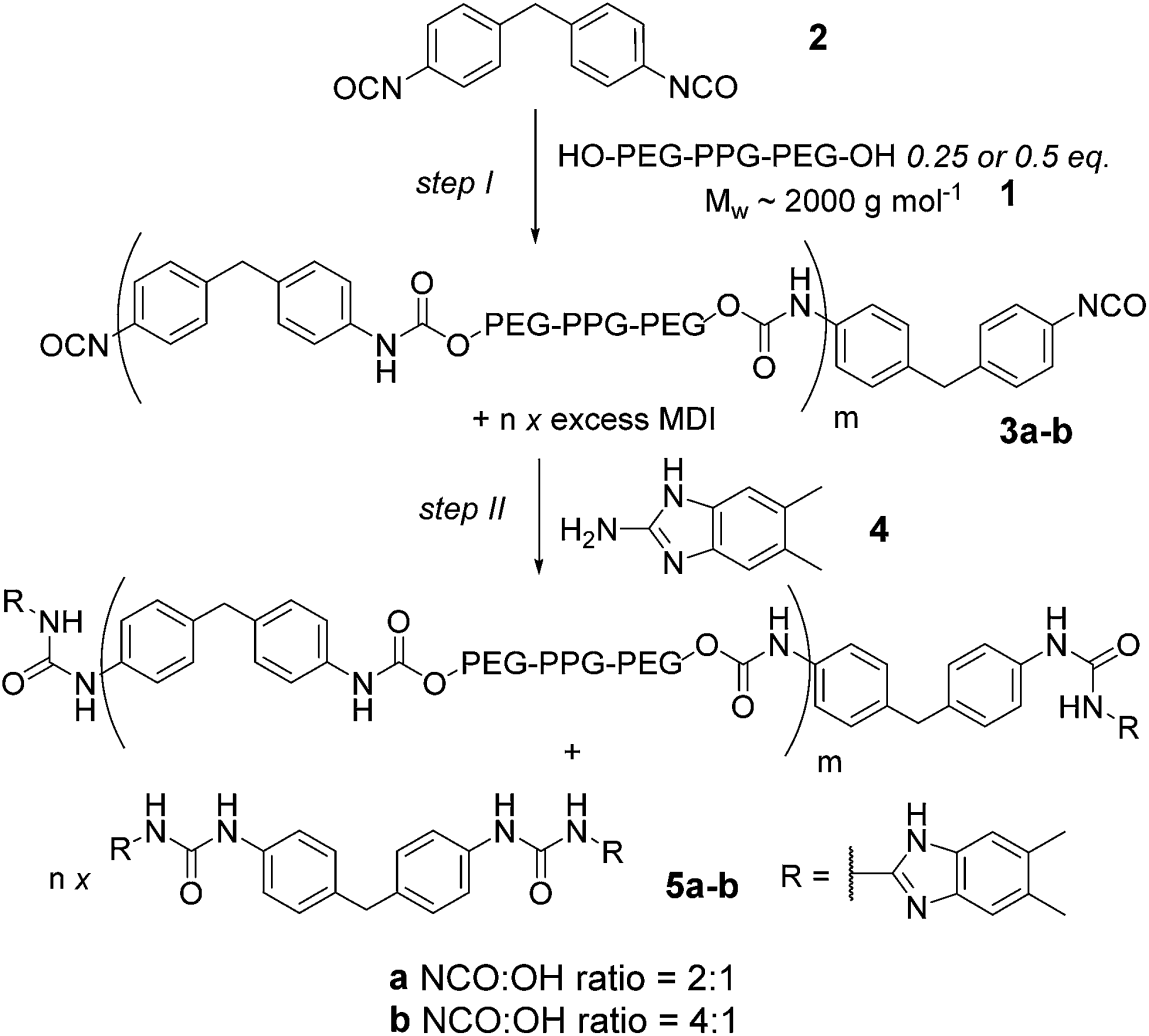
Solvent free synthesis of a supramolecular macromonomer by ball milling.

Milling methods were then used for synthesis of supramolecular polyurethanes **7** starting from MDI **2** and diol terminated PEG–PPG–PEG **1** (2000 g mol^–1^) (Scheme 3, step I). We focused on generating supramolecular polymers **7a–b** with a 2 : 1 and 4 : 1 NCO : OH ratio as in our previous work.^31^ A key feature of this reaction is its heterogeneity; the ditopic nature of each component results in polyurethanes **3a/b** (and thus **5a/b**) comprising a statistical mixture of capped and chain extended polyol **1** obtained alongside MDI **2** capped at both termini with the target amine (for **3/5a** *i.e.* NCO to OH ratio of 2 : 1, 50% of polyol can be excepted to be capped at both ends with MDI **2** whereas for **3/5b** *i.e.* NCO to OH ratio of 4 : 1 75% becomes capped). DAC **6** is then added to match the stoichiometry of the ureidoimidazole hydrogen-bonding motifs present in the system resulting in hydrogen-bond-mediated assembly of an elastomer in which the chain extended telechelic forms the soft block and the end capped MDI integrates into the hard-block with the DAC **6** resulting in different materials properties for different NCO to OH ratios.^31^

Initial studies focused on identifying suitable conditions for synthesis of **3a**. The reactants were subjected to milling at 20 Hz for three-minute intervals inside the ball mill. A test reaction with these conditions indicated urethane formation to be slower than carbamate formation. We therefore increased the vibrational frequency of milling to 25 Hz for five minute intervals for a total time of thirty minutes (Table 5). Macromonomer **3a** formation was found to depend upon the catalyst with DABCO the most proficient. This contrasts to carbamate formation in the model system. TBD in this case was also an effective catalyst, with a high crude conversion and purity, similar to that obtained using Et_3_N as catalyst (as evidenced by NMR) (Fig. S46†). Therefore further work is required to understand the complexity of the catalyst's interaction with polyols.

**Table 5:** Effect of catalyst on conversion of **2** to **3a** ^*a*^

Entry	Catalyst	p*K*_a_	Crude conversion/%
1	None	—	70
2	NEt_3_	10.8	78
3	DABCO	8.9	100
4	TBD	22	93

^*a*^Conditions: 10 mol% catalyst (entries 2–4) used, 0.5 equiv. of **1**, vibrational frequency was 25 Hz, 30 min. ^#^Conversion is based on comparison of NMR signals of crude reaction mixture.

**Fig. 3:**
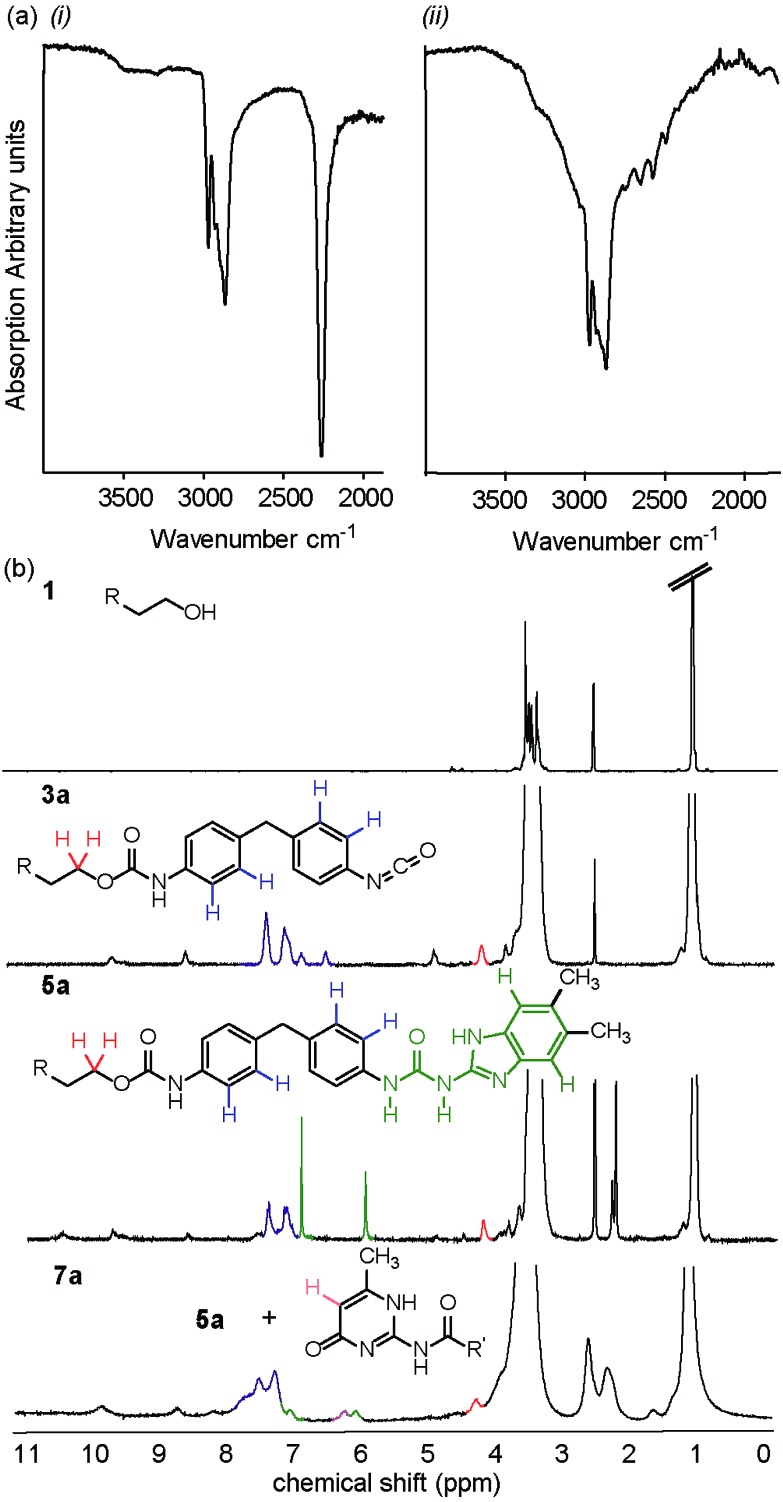
(a) IR spectrum showing reaction of the MDI end-capped PU (left), which shows free isocyanate at 2200 cm^–1^. Reaction with 2-amino-5,6-dimethylbenzimidazole shows that after ten minutes of milling (right), this absorption has disappeared suggesting urea formation with the amine. (b) ^1^H NMR spectra (300 MHz, DMSO-*d*_6_) of **1**, **3a**, **5a** and **7a** obtained by ball milling.

The macromonomer product **3a** was then reacted further with 2-amino-5,6-dimethylbenzimidazole **4** to produce the gummy, malleable supramolecular macromonomer **5a** (Scheme 3, step II). IR analysis of the material both before and after reaction (Fig. 3a) illustrated the disappearance of remaining isocyanate groups within 10 minutes. Similarly, NMR analyses of the synthetic sequence **1** to **3a** to **5a** illustrated formation of a carbamate as evidenced by the appearance of a resonance at 4.2 ppm expected for the OC*H*_2_–CO–NH group (Fig. 3b). Overall, this approach leads to an improved method: 10 mol% NEt_3_, DABCO or TBD (see Fig. S47†), 40 minutes at 25 Hz with no solvent relative to our original method (DMAc, 17.5 h at 87 °C).

We then applied our most suitable conditions to the synthesis and assembly of the supramolecular polyurethane elastomers **7a–b** in one pot using triethylamine as catalyst (Fig. 3b for **7a** and Fig. S48†); this was seen as advantageous because triethylamine can be removed under vacuum at the end of the procedure. The heterocomplementary triple hydrogen bonding array diamidoisocytosine (DAC) **6** could be added and the reaction subjected to further milling until a homogeneous tacky powder was achieved (∼20 min).

**Fig. 4:**
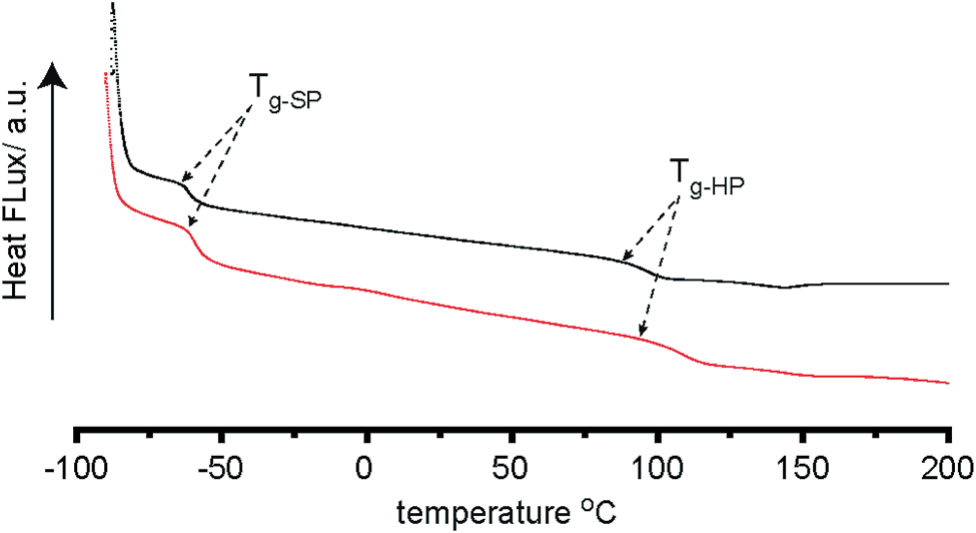
DSC traces of MDI and PEG–PPG–PEG based supramolecular polymer (4 : 1 NCO : OH ratio) synthesised by traditional solution (red) and solventless mechanochemistry in a ball mill (black).

Supramolecular polymer **7a–b** formation was assessed by differential scanning calorimetry (DSC) (Fig. 4 and S49†) which revealed defined glass transitions anticipated for an elastomeric material. For **7b** a broad exotherm shown at ∼ –61 °C can be attributed to the glass transition of the PEG based polymer backbone. A further transition at ∼97 °C can be attributed to transitions in the hydrogen bond containing hard blocks of the polymer. **7a** exhibits these transitions at ∼–61 °C and 120 °C respectively. These properties match those observed for supramolecular polymers prepared using the previously reported method shown in Fig. 1.^31^

## Conclusions

A systematic study on catalyst, temperature, solvent and reaction concentration has been performed to optimize the synthesis of carbamate, urethane and urea functional groups. This was compared against a solvent free method for this synthesis that exploits ball milling and which we developed in parallel. The method was found to be applicable to a wide range of substrates for small molecule synthesis and sufficiently efficient to proceed without catalyst. It should be noted that an increase in concentration was found to be detrimental in many cases as a result of gelation whereas no such problems were observed with ball milling. Most significantly we applied the method to the synthesis of a previously described supramolecular polyurethane **7a–b**. When compared to the optimized solution procedure, synthesis was shown to be faster and cleaner. Ultimately, the method could find use in development of synthetic processes with a reduced environmental impact and could operate in tandem with phosgene-free routes to isocyanates.^35^ Whilst mechanochemical synthesis of MOFs and crystalline frameworks is well established,^12,13^ synthesis of soft materials using this method is less prevalent; this makes the observation that supramolecular ordering and phase separation can be achieved during solvent free synthesis all the more remarkable, hence the results reported here may have more widespread applicability to other supramolecular polymer syntheses.
