# Supplementary material for: Development of solvent-free synthesis of hydrogen-bonded supramolecular polyurethanes
Source: Chem Sci. 2015 Feb 3;6(4):2382–8. doi: 10.1039/c4sc03804e (PMC5647484; doi:10.1039/c4sc03804e)
Supplement: Supplementary file 1 [file SC-006-C4SC03804E-s001.pdf]

Electronic Supplementary Material (ESI) for Chemical Science.
This journal is © The Royal Society of Chemistry 2015

Solvent Free Synthesis of Supramolecular Polyurethanes

Kelly A. Houton,[a] George M. Burslem[a,b] and Andrew J. Wilson[a,b]

[a] *School of Chemistry, University of Leeds, Leeds LS2 9JT, UK*
[b] *Astbury Centre for Structural Molecular Biology, University of Leeds, Leeds LS2 9JT, UK*

Electronic Supporting Information

Contents

GENERAL EXPERIMENTAL METHODS 3

General procedure for solution based carbamate synthesis 4

General procedure for solid phase carbamate synthesis 4

Synthesis of PEG-PPG-PEG bis(4-(4-isocyanatobenzyl)phenylcarbamate) 3a 4

Synthesis of PEG-PPG-PEG bis(4-(4-(3-(5,6-dimethyl-1H-benzo[d]imidazol-2-yl)ureido)benzyl)phenylcarbamate) 5a and Supramolecular polymer 7a and 7b 5

***N*1, *N8-bis*(6-methyl-4-oxo-1,4-dihydropyrimidin-2-yl)octanediamide, 6** 6

Dipropyl 4,4'-methylenebis(4,1-phenylene)dicarbamate 9a 6

- Fig. S1- catalyst effect in DMAc at 40 °C for 6hr. 8
- Fig. S2- demonstrating catalyst effect in toluene at room temperature for 6hr 9
- Fig. S3- demonstrating catalyst effect in toluene at 40 °C for 6hr. 10
- Fig. S4- Reaction of MDI 2 with 1-propanol 8a in toluene at 40 °C for 6 hours without catalyst 11
- Fig. S5- Reaction of MDI 2 with 1-propanol 8a in toluene at 40 °C for 6 hours with triethylamine catalyst 11
- Fig. S6- Reaction of MDI 2 with 1-propanol 8a in toluene at 40 °C for 6 hours with DABCO catalyst 12
- Fig. S7- Reaction of MDI 2 with n-propanol 8a in toluene at 40 °C for 6 hours with TBD catalyst 12
- Fig. S8- Isolated side product from the reaction of MDI 2 with 1-propanol 8a. Carbamate protons are identified in red, urea protons are identified in blue. Purple stars indicate starting material, which was inseparable from the side product. 13
- Fig. S9- Overlaid partial IR spectra of the reaction between MDI 2 and 1-propanol 8a over a time period of 6hr in toluene at 40 °C with Et_3N catalysis. The strong peak at 2200cm^{-1} which decreases periodically with time can be attributed to loss of free –NCO functionality. 13
- Fig. S10- demonstrating solvent effect on conversion to carbamate with Et_3N catalyst . 14
- Fig. S11- demonstrating effect of the concentration of the reaction Et_3N catalysis in toluene at 40 °C for 6hr 15
- Fig. S12- demonstrating catalyst effect in toluene at room temperature for 6hr 16
- Fig. S13- Reaction of MDI 2 with diethyleneglycol monoethyl ether 8b in toluene for 6 hours at r.t. with no catalyst 17

Fig. S14- Reaction of MDI 2 with diethyleneglycol monoethyl ether 8b in toluene for 6 hours at r.t. with triethylamine catalyst..17
Fig. S15- Reaction of MDI 2 with diethyleneglycol monoethyl ether 8b in toluene for 6 hours at r.t. with DABCO catalyst..18
Fig. S16- Reaction of MDI 2 with diethyleneglycol monoethyl ether 8b in toluene for 6 hours at r.t. with TBD catalyst..18
Fig. S17- demonstrating catalyst effect in toluene at room temperature for 6hr 19
Fig. S18- Reaction of MDI 2 with diethyleneglycol monoethyl ether 8b in toluene for 6 hours 40 °C with no catalyst..20
Fig. S19- Reaction of MDI 2 with diethyleneglycol monoethyl ether 8a in toluene for 6 hours at 40 °C with triethylamine catalyst 20
Fig. S20- Reaction of MDI 2 with diethyleneglycol monoethyl ether 8b in toluene for 6 hours at 40 °C with DABCO catalyst 21
Fig. S21- Reaction of MDI 2 with diethyleneglycol monoethyl ether 8b in toluene for 6 hours at 40 °C with TBD catalyst 21
Fig. S22- Overlaid partial IR spectra of the reaction between MDI 2 and diol terminated PEG-PPG-PEG 1 in toluene at 40 °C with Et_3N catalysis for 6hr.....................................22
Fig. S23- the reaction between MDI 2 and diol terminated PEG-PPG-PEG 1 in toluene at room temperature..22
Fig. S24- reaction between MDI 2 and diol terminated PEG-PPG-PEG 1 in toluene at 40 °C..23
Fig. S25 - between MDI 2 and n-propanol 8a in the ball mill at 20 Hz for 18 minutes.....24
Fig. S26- Reaction of MDI 2 with 1-propanol 8a in the ball mill for 18 minutes at 20 Hz with no catalyst..24
Fig. S27- Reaction of MDI 2 with 1-propanol 8a in the ball mill for 18 minutes at 20 Hz with triethylamine catalyst..25
Fig. S28- Reaction of MDI 2 with 1-propanol 8a in the ball mill for 18 minutes at 20 Hz with DABCO catalyst..25
Fig. S29- Reaction of MDI 2 with 1-propanol 8a in the ball mill for 18 at 20 Hz minutes with TBD catalyst..26
Fig. S30 - between MDI 2 and diethyleneglycol monoethyl ether 8b in the ball mill at 20 Hz for 18 minutes. 27
Fig. S31- Reaction of MDI 2 with diethyleneglycol monoethyl ether 8b in the ball mill for 18 minutes at 20 Hz with no catalyst..28
Fig. S32- Reaction of MDI 2 with diethyleneglycol monoethyl ether 8b in the ball mill for 18 minutes at 20 Hz with triethylamine catalyst 28
Fig. S33- Reaction of MDI 2 with diethyleneglycol monoethyl ether 8b in the ball mill for 18 minutes at 20 Hz with DABCO catalyst 29
Fig. S34- Reaction of MDI 2 with diethyleneglycol monoethyl ether 8b in the ball mill for 18 at 20 Hz minutes with TBD catalyst 29
Fig. S35- reaction of p-cyano phenylisocyanate with 2-aminobenzimidazole in the ball mill for 10 minutes to give 11a..30
Fig. S36- reaction of p-nitro phenylisocyanate with diisopropylamine in the ball mill for 10 minutes to give 11b. 30
Fig. S37 Reaction of p-ethyl phenylisocyanate with diisopropylamine in the ball mill for 10 minutes to give 11c 31
Fig. S38- Reaction of adamantyl isocyanate with 2-aminobenzimidazole in the ball mill for 10 minutes to give 11d. Crude conversion was taken from integration of product protons (starred) against starting material (DMSO-d_6, 500 MHz). 31
Fig. S39- Reaction of adamantyl isocyanate with diisopropylamine in the ball mill for 10 minutes to give 11e 32

Fig. S40- Reaction of p-nitro phenylisocyanate with n-propanol in the ball mill for 10 minutes to give 12a. 32
Fig. S41- Reaction of adamantyl isocyanate with n-propanol in the ball mill for 10 minutes to give 12b. 33
Fig. S42- Reaction of adamantyl isocyanate with O-nitro benzylalcohol in the ball mill for 10 minutes to give 12c 33
Fig. S43- Reaction of p-cyano phenylisocyanate with o-nitrobenzyl alcohol in the ball mill for 10 minutes to give 12d. 34
Fig. S44 Reaction of p-nitro phenylisocyanate with o-nitro benzylalcohol in the ball mill for 10 minutes to give 12e. 34
Fig. S45- Reaction of p-ethyl phenylisocyanate with o-bromo benzylalcohol in the ball mill for 10 minutes to give 12f. 35
Fig. S46- Demonstrating catalyst effect on polyurethane 3a formation in the ball mill for 30min 36
Fig. S47 Demonstrating catalyst effect on macromonomer, 5a formation in the ball mill for 10min..37
Fig. S48 Full spectrum of supramolecular polymers, 7a and 7b, formed in the ball mill. 38
Fig. S49 Differential Scanning Calorimetry (DSC) scans of supramolecular polymers formed synthesized in a ball mall..39

GENERAL EXPERIMENTAL METHODS

All reactions were performed under a nitrogen atmosphere unless otherwise stated. Reagents were purchased from major suppliers and used without further purification unless otherwise stated. Anhydrous toluene, hexane, diethyl ether, tetrahydrofuran and chloroform were obtained from the in-house solvent purification system from Innovative Inc. PureSolv®. Anhydrous dimethylacetamide and 1,4-dioxane were obtained from Sigma Aldrich equipped with Sure/Seal™. Triethylamine was distilled from calcium hydride and was stored under nitrogen over potassium hydroxide pellets prior to use. 1-Propanol was distilled from calcium hydride directly before use. Poly(ethylene glycol)-*block*-poly(propylene glycol)-*block*-poly(ethylene glycol) was heated at 60°C, over 4Å molecular sieves under vacuum for 6 hours prior to use. DABCO was recrystallized from petroleum ether and dried before use. Prior to use, TBD was dried by stirring in tetrahydrofuran with calcium hydride, filtering and removing the solvent *in vacuo*. Analytical thin layer chromatography was performed on Merck Kiesegel 60 F_{254} 0.25 mm pre-coated aluminium plates. Product spots were visualised under UV light (λ_{max} = 254 nm). Flash chromatography was carried out using Merck Kieselgel 60 silica gel. Nuclear magnetic resonance spectra were obtained using a Bruker AMD300 or Bruker DMX500 spectrometer operating at 300 MHz or 500 MHz for ^{1}H spectra and 75 MHz or 125 MHz for ^{13}C spectra as stated. Reactions followed by infra-red spectroscopy used a Perkin-Elmer FTIR spectrometer where absorption maxima (υ_{max}) are quoted in wavenumbers (cm^{-1}) and only structurally relevant absorptions have been included. High Resolution Mass Spectra (HRMS) were recorded on a Bruker Daltonics microTOF using electrospray ionisation (ESI).

General procedure for solution based carbamate synthesis

The alcohol (2.0 mmol) was stirred in the required dry solvent (20 mL) at the required temperature. The required amount of catalyst (10 mol%) was added in one portion and stirred for a further 10 minutes. Methylene diphenyl diisocyanate (0.25 g, 1.0 mmol) was added in one portion and the reaction mixture was stirred for a further 6 hours before removal of the solvent *in vacuo*. The recovered solid was purified by column chromatography.

General procedure for solid phase carbamate synthesis

The alcohol (1.85 mmol), the required amount of catalyst (10 mol%) and the isocyanate or diisocyanate (1.85 mmol or 0.93 mmol) were added in one portion to the reaction cups (stainless steel, 10 mL) and the reaction mixture was ball-milled for 3 minute intervals at 20 Hz. The recovered solid was subjected to crude ^{1}H NMR and LC-MS analysis. The Retsch Mixer Mill 200 was supplied by Retsch.

Synthesis of PEG-PPG-PEG bis(4-(4-isocyanatobenzyl)phenylcarbamate) 3a

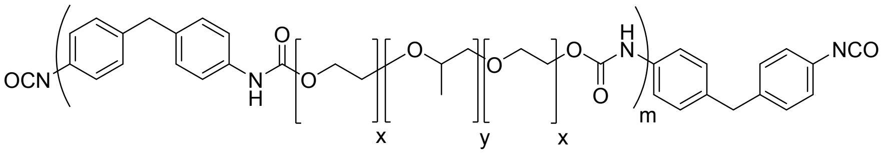

Solution Poly(ethylene glycol)-*block*-poly(propylene glycol)-*block*-poly(ethylene glycol) (2 mL, 1 mmol) was stirred in toluene (20 mL) at the required temperature. The required amount of catalyst (10 mol%) was added in one portion and stirred for a further 10 minutes. MDI (0.5 g, 2 mmol) was added in one portion and the reaction mixture was stirred for a further 6 hours before removal of the solvent *in vacuo.* The recovered solid was subjected to crude NMR analysis only.

Bulk Poly(ethylene glycol)-*block*-poly(propylene glycol)-*block*-poly(ethylene glycol) (2 mL, 1 mmol), the required amount of catalyst (10 mol%) and MDI (0.5 g, 2 mmol) were added in one portion to the reactions cups (stainless steel, 10 mL) and the reaction mixture was milled for 5 minute intervals at 25 Hz, at which point small aliquots were removed for IR analysis. The recovered solid was subjected to crude NMR analysis to confirm identity of the material and conversion.

Synthesis of PEG-PPG-PEG bis(4-(4-(3-(5,6-dimethyl-1H-benzo[d]imidazol-2-yl)ureido)benzyl)phenylcarbamate) 5a and Supramolecular polymer 7a and 7b

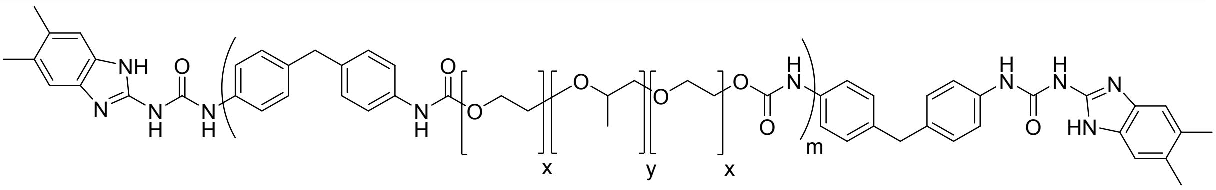

Solution synthesis of **7a** and **7b** was performed as described previously[1]

Bulk for 7a 5,6-dimethyl 2-aminobenzimidazole **4** (0.32 g, 2 mmol) was added to the product **3** obtained from the reaction of Poly(ethylene glycol)-*block*-poly(propylene glycol)-*block*-poly(ethylene glycol) **1** with MDI **2** to the reactions cups (stainless steel, 10 mL) and the reaction mixture was milled for 5-minute intervals at 25 Hz. After satisfaction of NCO stretches by IR, the diotpic DAC **6** was added in one portion and the reaction mixture further milled at 5 min intervals for 20 min until a homogenous sample was obtained which was dried further *in vacuo* and subjected to NMR and IR analysis to confirm identity of the material.

Bulk for 7b was obtained using the procedure above for **7a** and the appropriate stoichiometries of reagents

*N*1, *N8-bis*(6-methyl-4-oxo-1,4-dihydropyrimidin-2-yl)octanediamide, 6

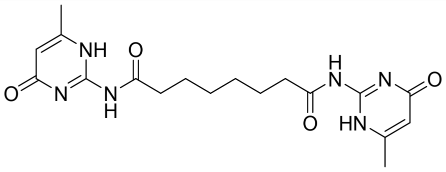

Synthesised using minor modifications to a literature procedure.[2] 2-Amino-4-hydroxy-6-methylpyrimidine (5.99 g, 48 mmol) was dissolved in dimethylacetamide (DMAc) (50 mL) at 87 °C. The reaction mixture was allowed to cool to room temperature then triethylamine (6.65 mL, 48 mmol) was added dropwise with stirring. After 10 minutes, suberoyl chloride (4.33 mL, 24 mmol) was added dropwise at 0 °C. Subsequently the reaction mixture was heated to 87 °C and was left to stir for 16 hr. The reaction mixture was allowed to cool to room temperature and the solvent was removed *via* vacuum distillation. The crude product was triturated (H_2O) followed by recrystallisation $CHCl_3$ – hexane to provide the title compound 86% (8.03 g) as a colourless powder. Characterisation was consistent with that reported previously.[2]

Dipropyl 4,4'-methylenebis(4,1-phenylene)dicarbamate 9a

1-Propanol (0.15 mL, 2 mmol) was stirred in the required dry solvent (20 mL) at the required temperature. The required amount of catalyst (10 mol%) was added in one portion and stirred for a further 10 minutes. MDI (0.25 g, 1 mmol) was added in one portion and the reaction mixture was stirred for a further 6 hours before removal of the solvent *in vacuo*. The recovered solid was purified by column chromatography (1:19 MeOH−CH_2Cl_2) to give the *title compound* (64%) as a colourless powder; δ_H (300 MHz, $CDCl_3$): 7.20 (4H, d, *J*= 8.6 Hz, Ar-H), 7.02 (4H, d, *J*= 8.6 Hz, Ar-H), 6.46 (2H, bs, N-H), 4.04 (4H, t, *J*=6.7 Hz, O-$C\underline{H_2}CH_2CH_3$), 3.84 (2H, s, methylene-$C\underline{H_2}$), 1.63 (4H, app hextet, *J*=7.1 Hz, O-$CH_2C\underline{H_2}CH_3$), 0.92 (6H, t, *J*=7.5 Hz, O-$CH_2CH_2C\underline{H_3}$); δ_C (75 MHz, $CDCl_3$): 136.5, 136.0,

118.9, 118.8, 80.1, 66.8, 40.5, 22.3 and 10.4; ν_{max}/cm^{-1} (solid state) =3405, 1739, 1523 and 1204; ESI-HRMS found mass 393.1794 $[M + Na]^+$ $C_{21}H_{26}N_2O_4Na$ requires 393.1794.

Di-2-(2-ethoxyethoxy)ethyl 4,4'-methylenebis(4,1-phenylene)dicarbamate 9b

The compound was prepared following the same procedure as for **9a** on a 2 mmol scale. A sample was purified by column chromatography eluting with (2:98 MeOH-CH_2Cl_2) to give the *title compound* as a colourless oil; ^{1}H NMR (500 MHz, DMSO) δ 9.65 (s, 2H, carbamate NH), 7.38 (d, *J* = 8.6 Hz, 4H, Ar-H), 7.10 (d, *J* = 8.6 Hz, 4H, Ar-H), 4.23 – 4.15 (m, 4H, ethylene-CH_2), 3.80 (s, 2H, methylene-CH_2), 3.68 – 3.62 (m, 4H, ethylene-CH_2), 3.55 (dd, *J* = 5.9, 3.6 Hz, 4H, ethylene-CH_2), 3.49 (dd, *J* = 5.8, 3.6 Hz, 4H, ethylene-CH_2), 3.43 (q, *J* = 7.0 Hz, 4H, ethyl CH_2), 1.10 (t, *J* = 7.0 Hz, 6H, terminal CH_3); ^{13}C NMR (125 MHz, DMSO) δ 153.93, 137.54, 135.93, 129.28, 118.82, 70.26, 69.66, 69.17, 66.04, 63.90, 15.55; ν_{max}/cm^{-1} (solid state) = 3292, 2867, 1703, 1531; ESI-HRMS found mass 519.2705 $[M + H]^+$ $C_{27}H_{39}N_2O_8$ requires 519.2709.

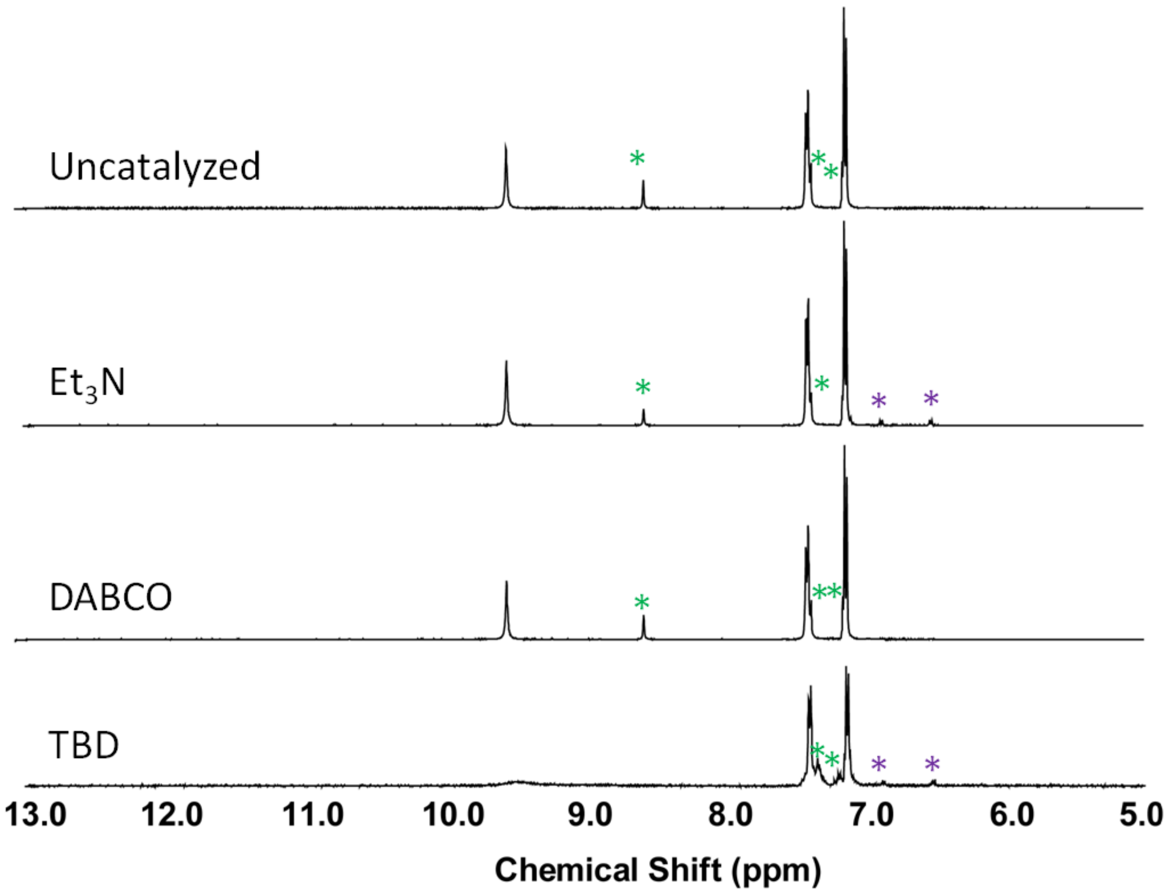

Fig. S1- catalyst effect in DMAc at 40 °C for 6hr. Partial crude NMR (DMSO-$d_{6,}$ 500 MHz);. Crude conversion was taken by integrating MDI protons in the desired product (doublets at 7.1ppm and 7.3ppm) against unreacted (6.5ppm and 6.8ppm) material. Reaction concentration 0.05 M, 10 mol% catalyst used, 1 equiv. of **2**, 2 equiv. of **8a**, toluene, 6h. * indicative of MDI urea side product – see fig. S8, * indicative of starting material

Catalyst	Conversion to Carbamate/ %
**Uncatalyzed**	80
**Triethylamine**	88
**DABCO**	82
**TBD**	0

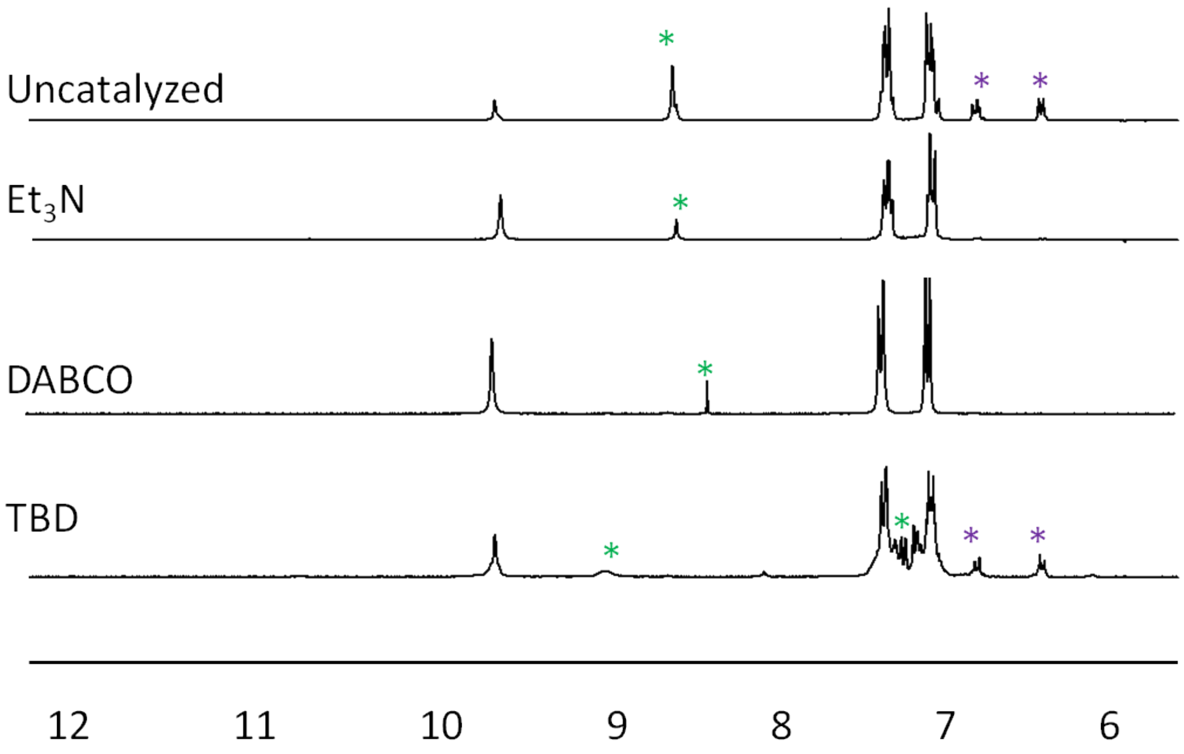

Fig. S2- demonstrating catalyst effect in toluene at room temperature for 6hr. Partial crude NMR (DMSO-d_6, 500 MHz); Crude conversion was taken by integrating MDI protons in the desired product (doublets at 7.1ppm and 7.3ppm) against unreacted (6.5ppm and 6.8ppm) and side product material. Reaction concentration 0.05 M, 10 mol% catalyst used, 1 equiv. of **2**, 2 equiv. of **8a**, toluene, 6h.* indicative of MDI urea side product– see fig. S8, * indicative of starting material

Catalyst	Conversion to Carbamate/ %
**Uncatalyzed**	29
**Triethylamine**	75
**DABCO**	91
**TBD**	67

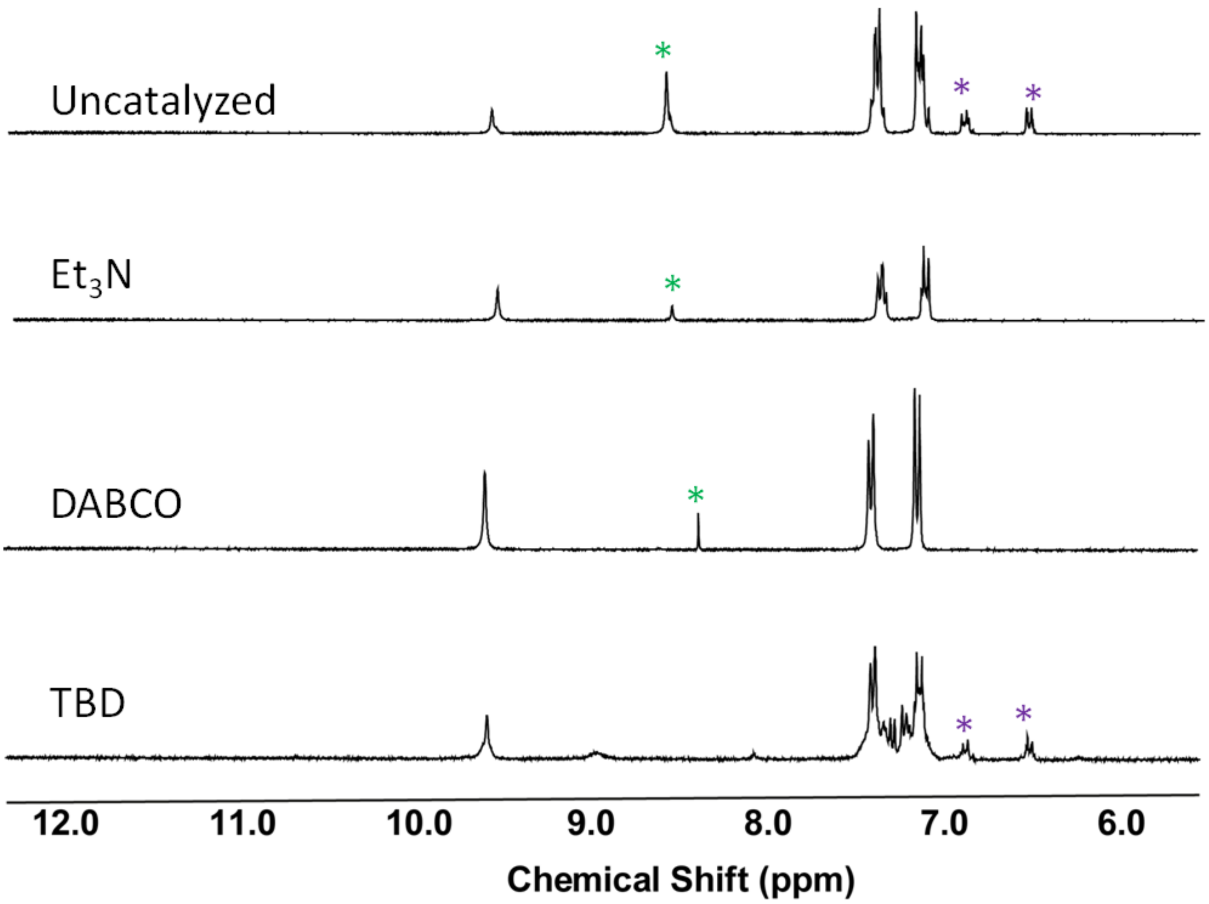

Fig. S3- demonstrating catalyst effect in toluene at 40 °C for 6hr. Partial crude NMR (DMSO-d_6, 500 MHz); Crude conversion was taken by integrating MDI protons in the desired product (doublets at 7.1ppm and 7.3ppm) against unreacted (6.5ppm and 6.8ppm) material. Reaction concentration 0.05 M, 10 mol% catalyst used, 1 equiv. of **2**, 2 equiv. of **8a**, toluene, 6h. * indicative of MDI urea side product– see fig. S8, * indicative of starting material

Catalyst	Conversion to Carbamate/ %
**Uncatalyzed**	29
**Triethylamine**	74
**DABCO**	97
**TBD**	66

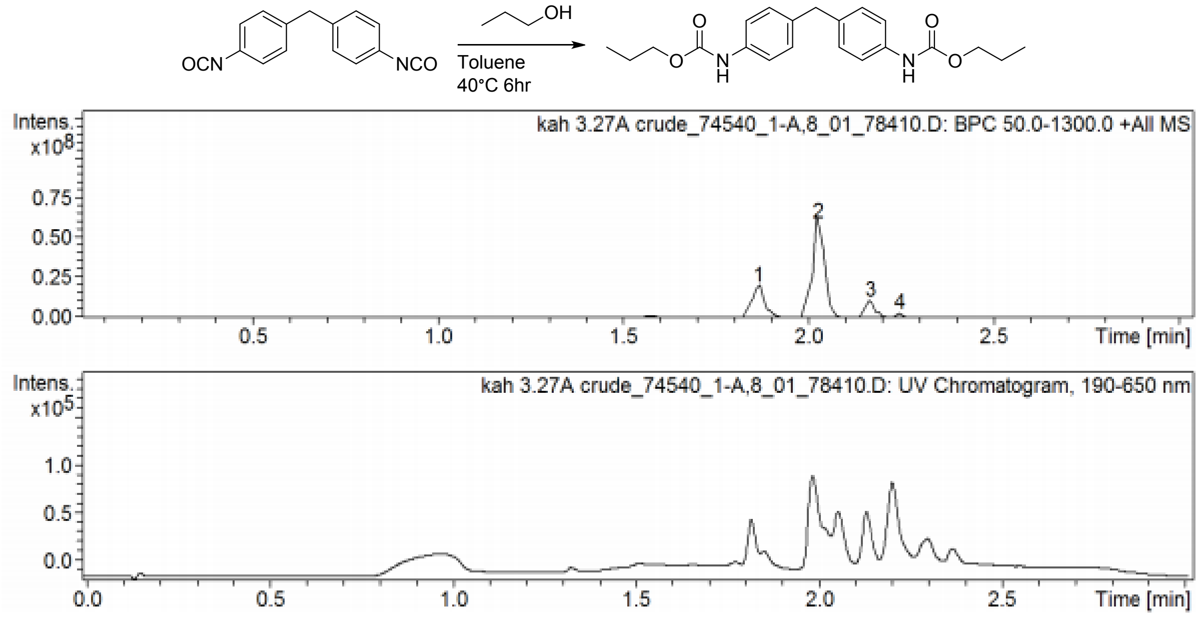

Fig. S4- Reaction of MDI **2** with 1-propanol **8a** in toluene at 40 °C for 6 hours without catalyst.

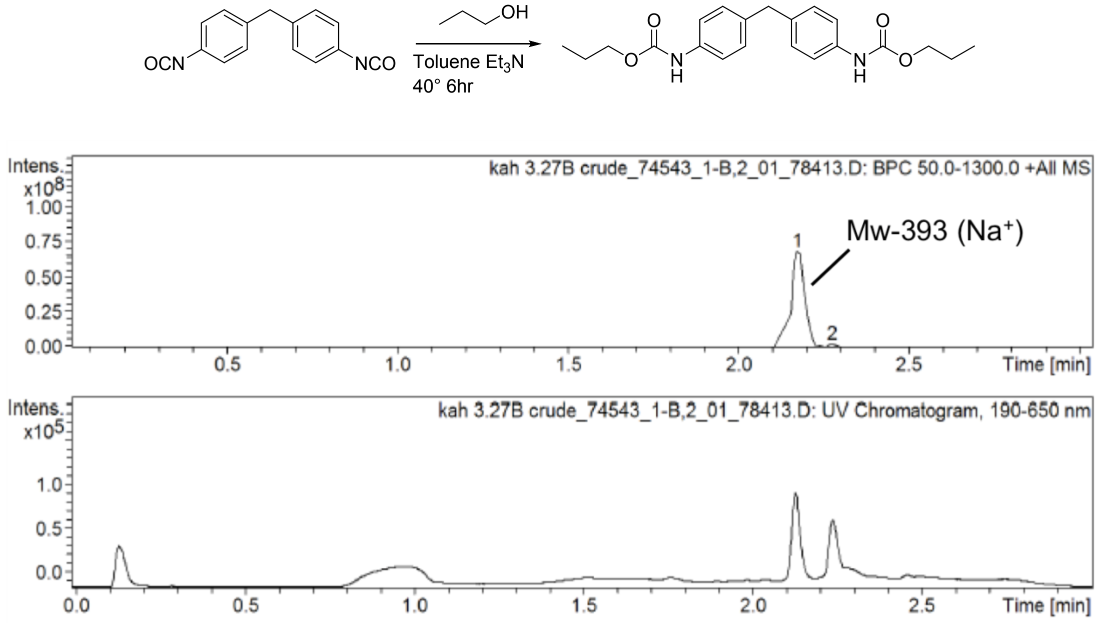

Fig. S5- Reaction of MDI **2** with 1-propanol **8a** in toluene at 40 °C for 6 hours with triethylamine catalyst. The peak labelled 1 is the desired product **9a**. The peak labelled 2 is the urea of MDI.

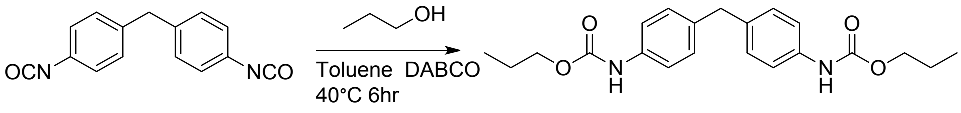

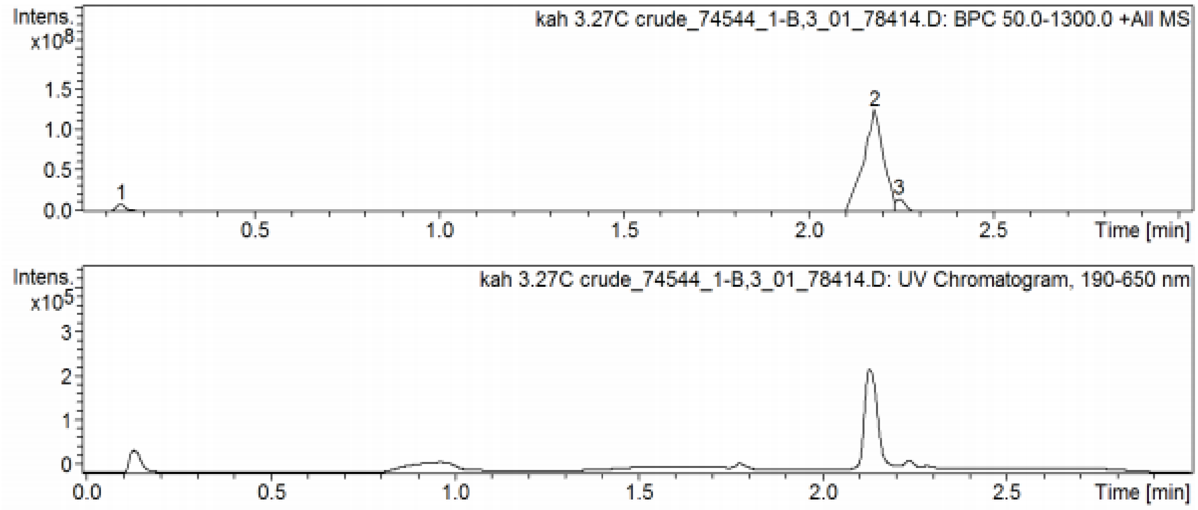

Fig. S6- Reaction of MDI **2** with 1-propanol **8a** in toluene at 40 °C for 6 hours with DABCO catalyst. The peak labelled 1 is the desired product **9a**. The peak labelled 2 is the urea of MDI.

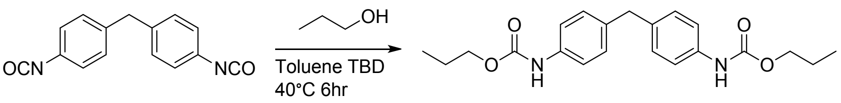

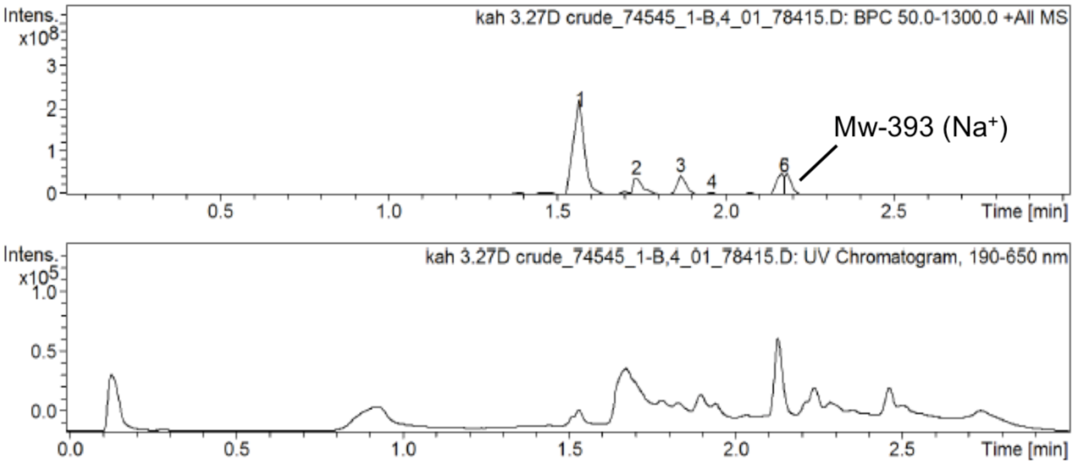

Fig. S7- Reaction of MDI **2** with n-propanol **8a** in toluene at 40 °C for 6 hours with TBD catalyst. The peak labelled 6 is the desired product **9a**.

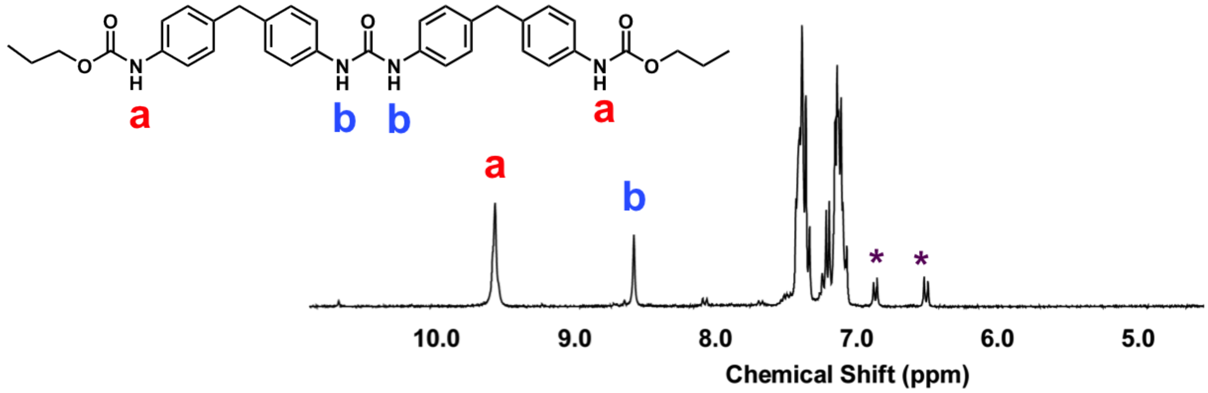

Fig. S8- Isolated side product from the reaction of MDI **2** with 1-propanol **8a.** Carbamate protons are identified in red, urea protons are identified in blue. Purple stars indicate starting material, which was inseparable from the side product.

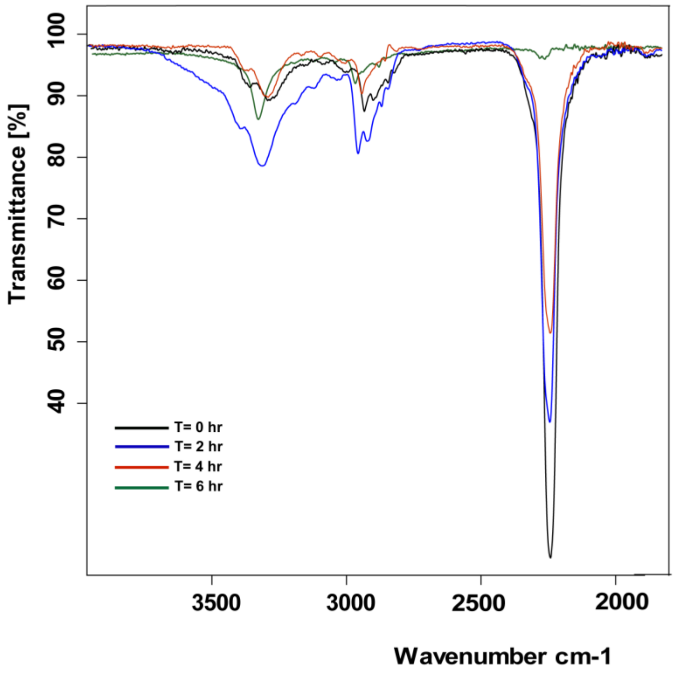

Fig. S9- Overlaid partial IR spectra of the reaction between MDI **2** and 1-propanol **8a** over a time period of 6hr in toluene at 40 °C with Et_3N catalysis. The strong peak at 2200cm^{-1} which decreases periodically with time can be attributed to loss of free –NCO functionality.

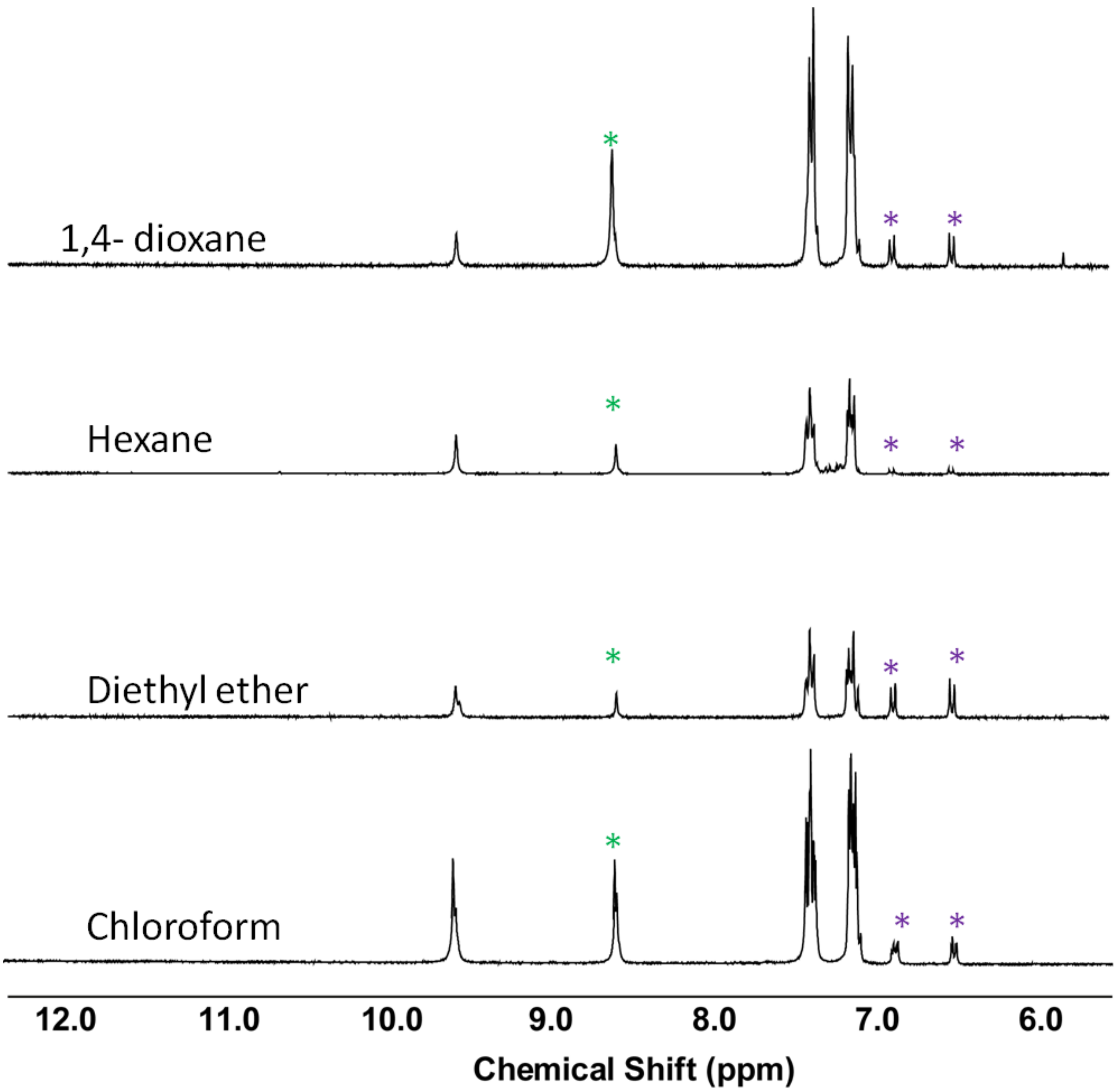

Fig. S10- demonstrating solvent effect on conversion to carbamate with Et_3N catalyst. Partial crude NMR (DMSO-d_6, 500 MHz); 10 mol% catalyst at 40 °C for 6hr. Crude conversion was taken by integrating MDI protons in the desired product (doublets at 7.1ppm and 7.3ppm) against unreacted (6.5ppm and 6.8ppm) material. Reaction concentration 0.05 M, 1 equiv. of **2**, 2 equiv. of **8a**, toluene, 6h. * indicative of MDI urea side product, * indicative of starting material

Solvent	Conversion to Carbamate/ %
**1,4-dioxane**	21
**Hexane**	60
**Diethyl Ether**	69
**Chloroform**	54

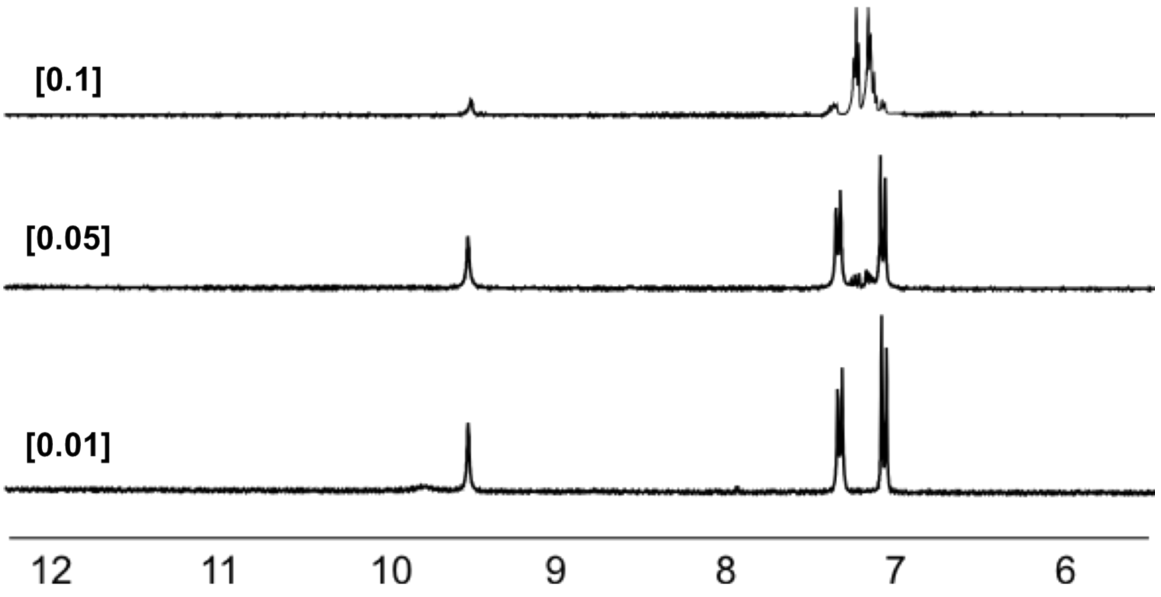

Fig. S11- demonstrating effect of the concentration of the reaction Et_3N catalysis in toluene at 40 °C for 6hr. Partial crude NMR (DMSO-d_6, 500 MHz). 1 equiv. of **2**, 2 equiv. of **8a**. Crude conversion was taken by integrating MDI protons in the desired product (doublets at 7.1ppm and 7.3ppm) against unreacted (6.5ppm and 6.8ppm) material.

Concentration/ M	Conversion to Carbamate/ %
**0.1**	12
**0.05**	87
**0.01**	100

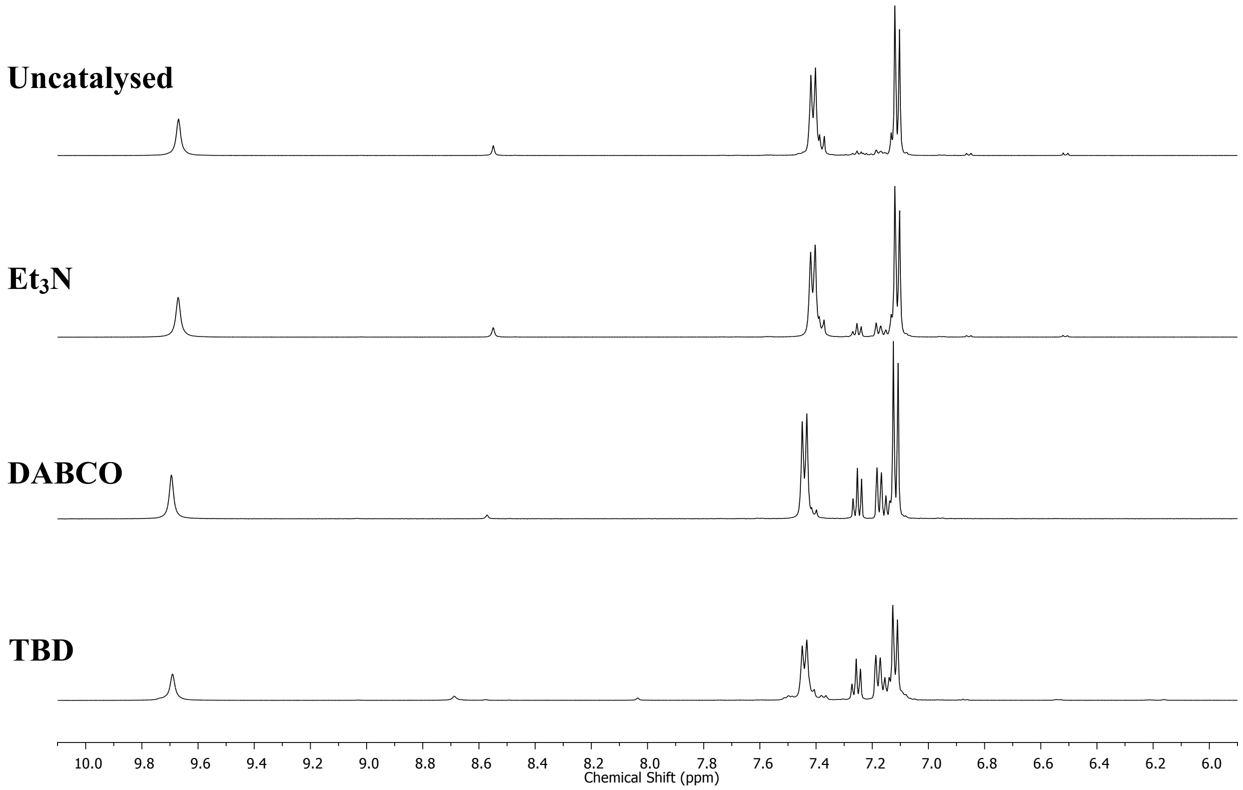

Fig. S12- demonstrating catalyst effect in toluene at room temperature for 6hr. Partial crude NMR (DMSO-d_6, 500 MHz); Crude conversion was taken by integrating MDI protons in the desired product (doublets at 7.1ppm and 7.3ppm) against unreacted (6.5ppm and 6.8ppm) and side product material. Reaction concentration 0.05 M, 10 mol% catalyst used, 1 equiv. of **2**, 2 equiv. of **8b**, toluene, rt.

Catalyst	Conversion to Carbamate/ %
**Uncatalyzed**	84
**Triethylamine**	84
**DABCO**	88
**TBD**	63

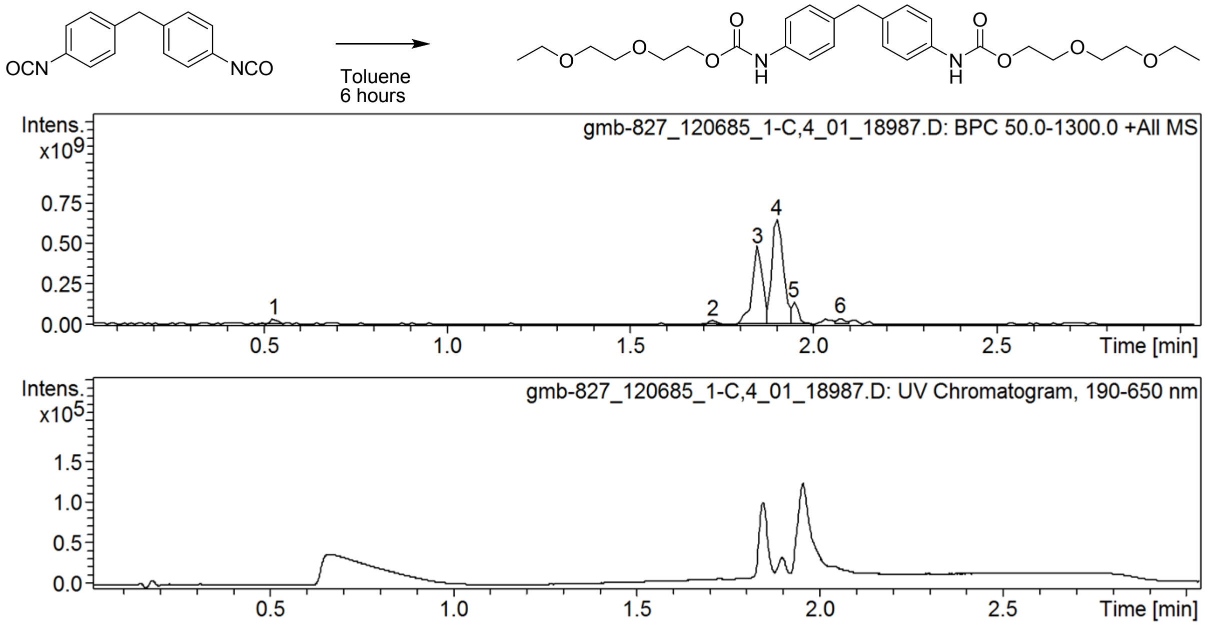

Fig. S13- Reaction of MDI **2** with diethyleneglycol monoethyl ether **8b** in toluene for 6 hours at r.t. with no catalyst

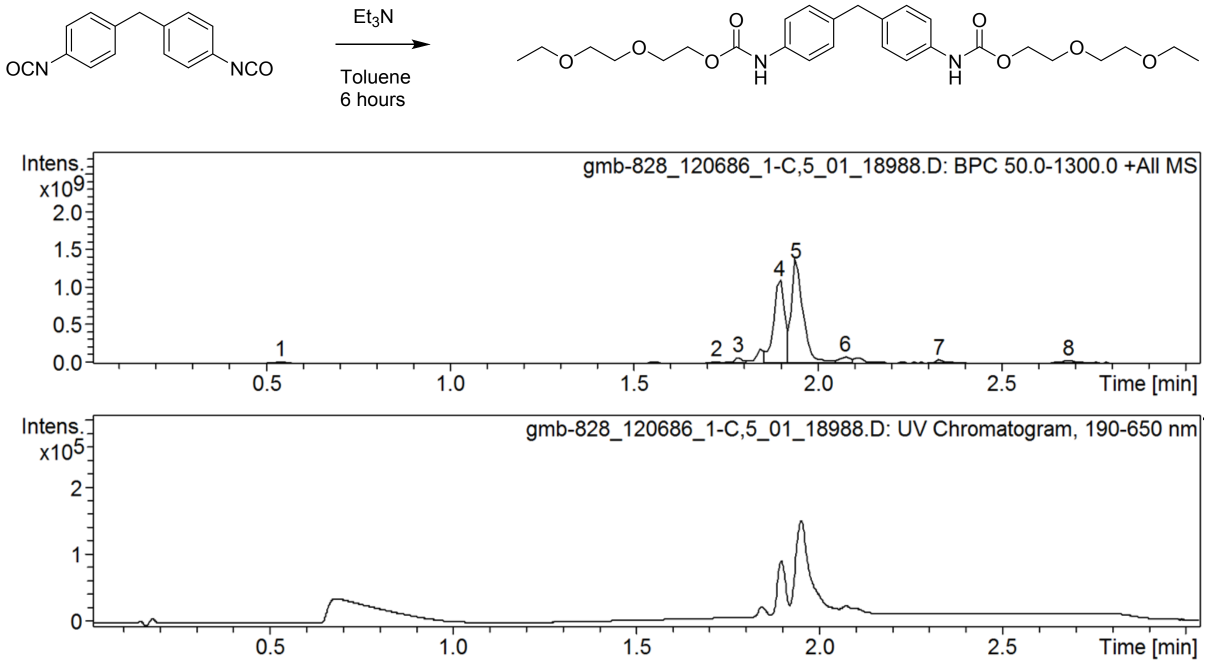

Fig. S14- Reaction of MDI **2** with diethyleneglycol monoethyl ether **8b** in toluene for 6 hours at r.t. with triethylamine catalyst

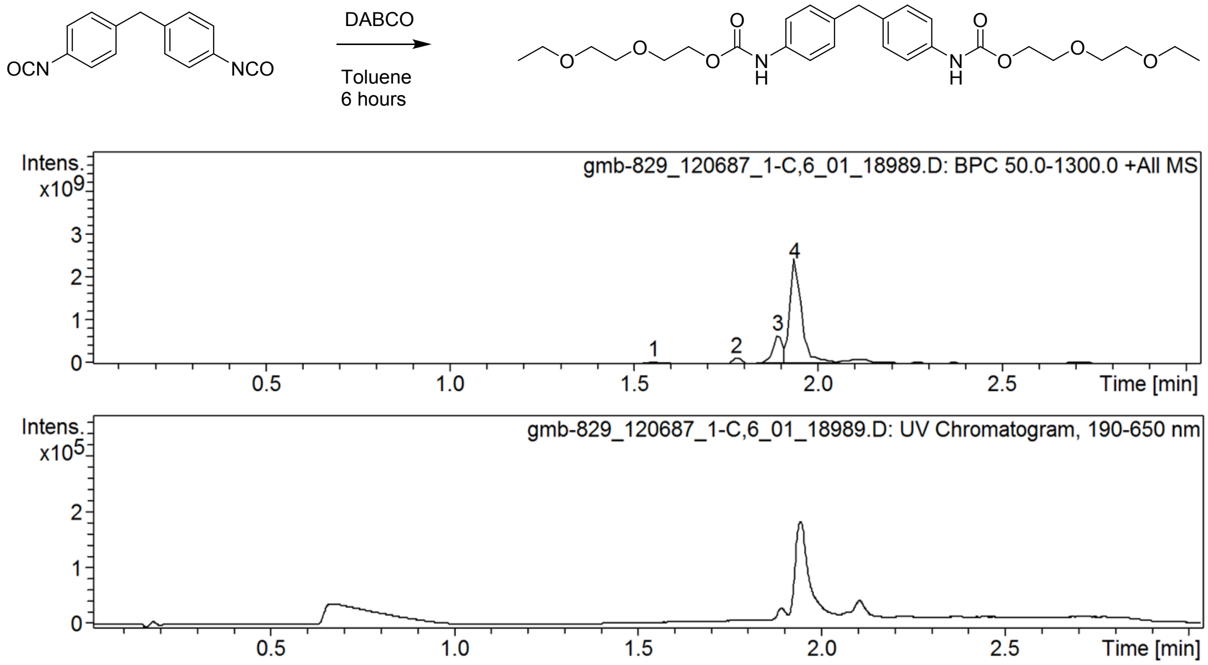

Fig. S15- Reaction of MDI **2** with diethyleneglycol monoethyl ether **8b** in toluene for 6 hours at r.t. with DABCO catalyst

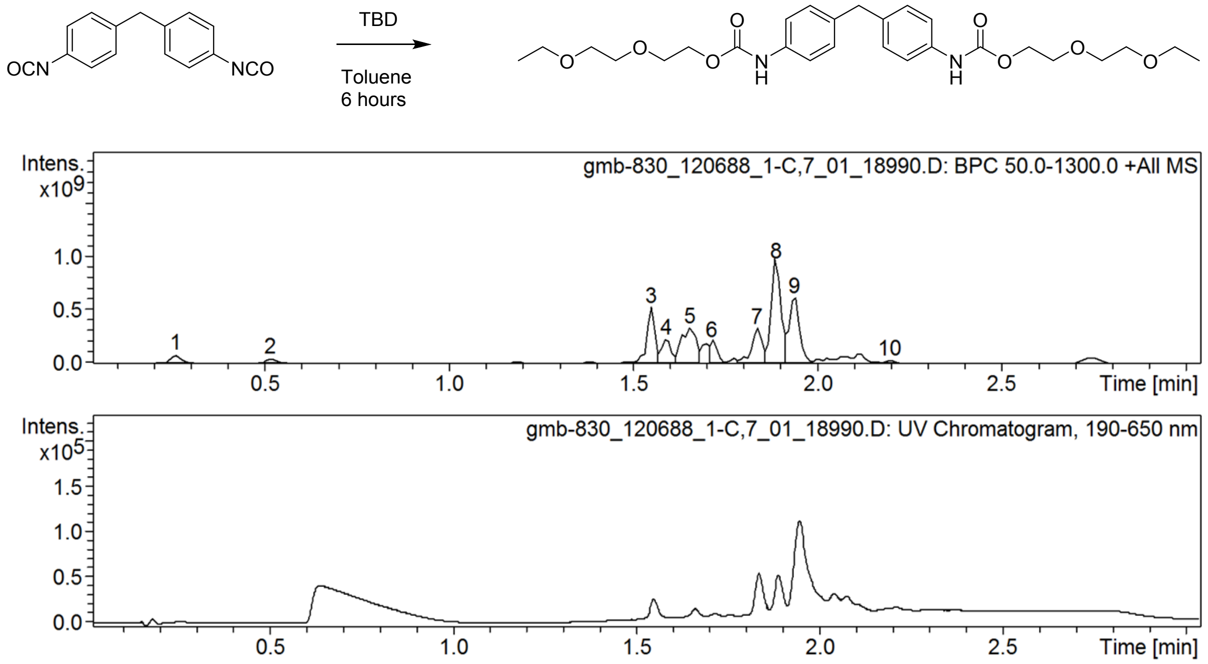

Fig. S16- Reaction of MDI **2** with diethyleneglycol monoethyl ether **8b** in toluene for 6 hours at r.t. with TBD catalyst

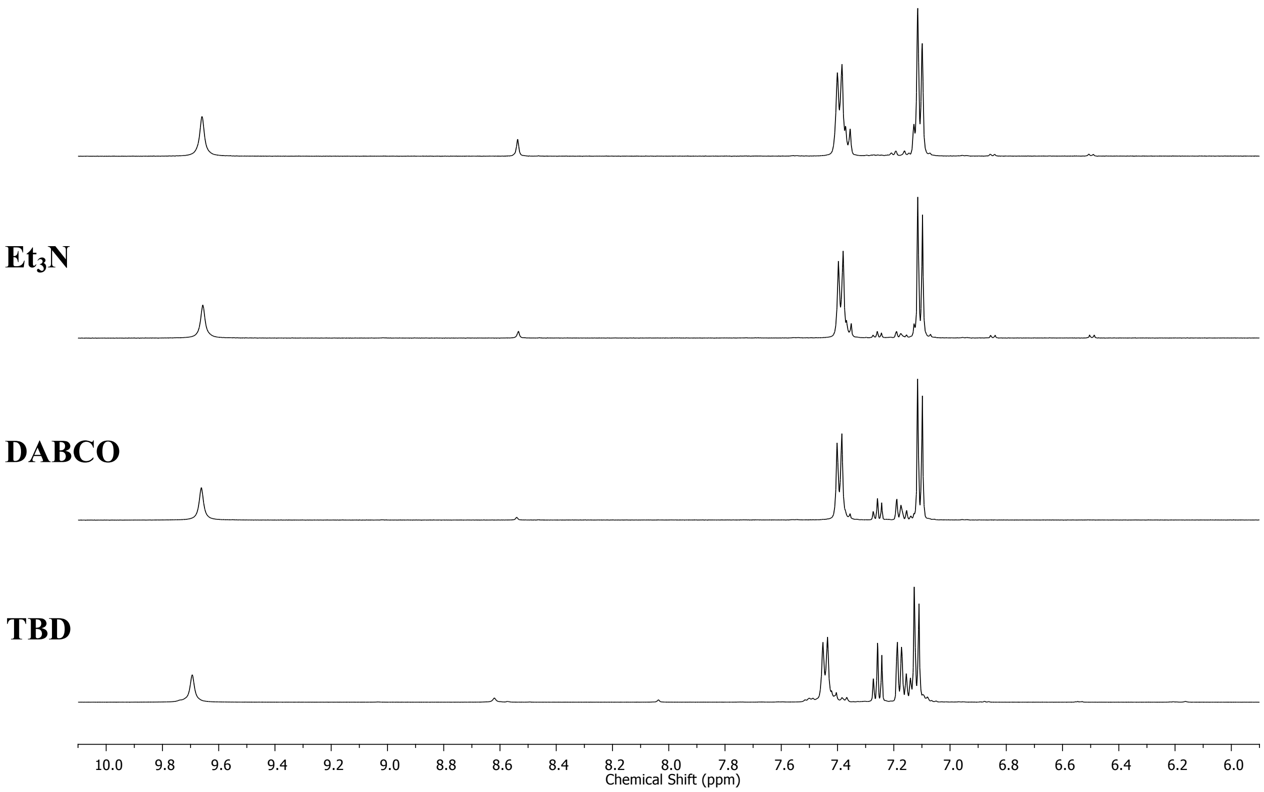

Fig. S17- demonstrating catalyst effect in toluene at room temperature for 6hr. Partial crude NMR (DMSO-d_6, 500 MHz); Crude conversion was taken by integrating MDI protons in the desired product (doublets at 7.1ppm and 7.3ppm) against unreacted (6.5ppm and 6.8ppm) and side product material. Reaction concentration 0.05 M, 10 mol% catalyst used, 1 equiv. of **2**, 2 equiv. of **8b**, toluene, 40°C.

Catalyst	Conversion to Carbamate/ %
**Uncatalyzed**	88
**Triethylamine**	89
**DABCO**	92
**TBD**	62

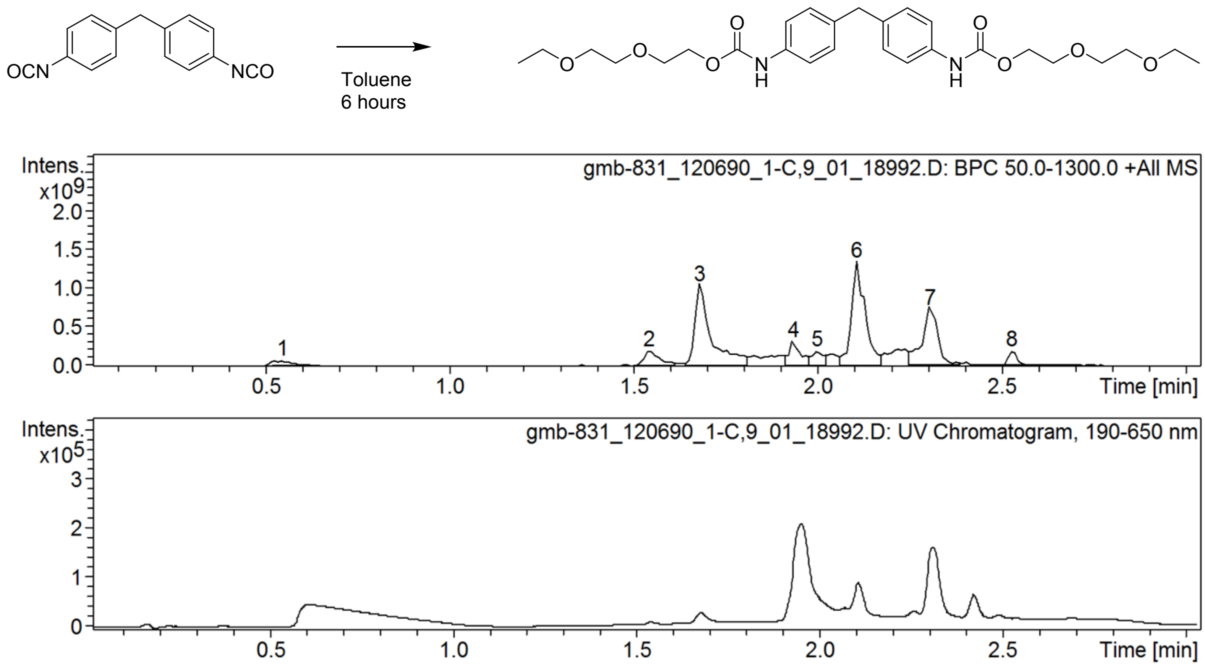

Fig. S18- Reaction of MDI **2** with diethyleneglycol monoethyl ether **8b** in toluene for 6 hours 40 °C with no catalyst

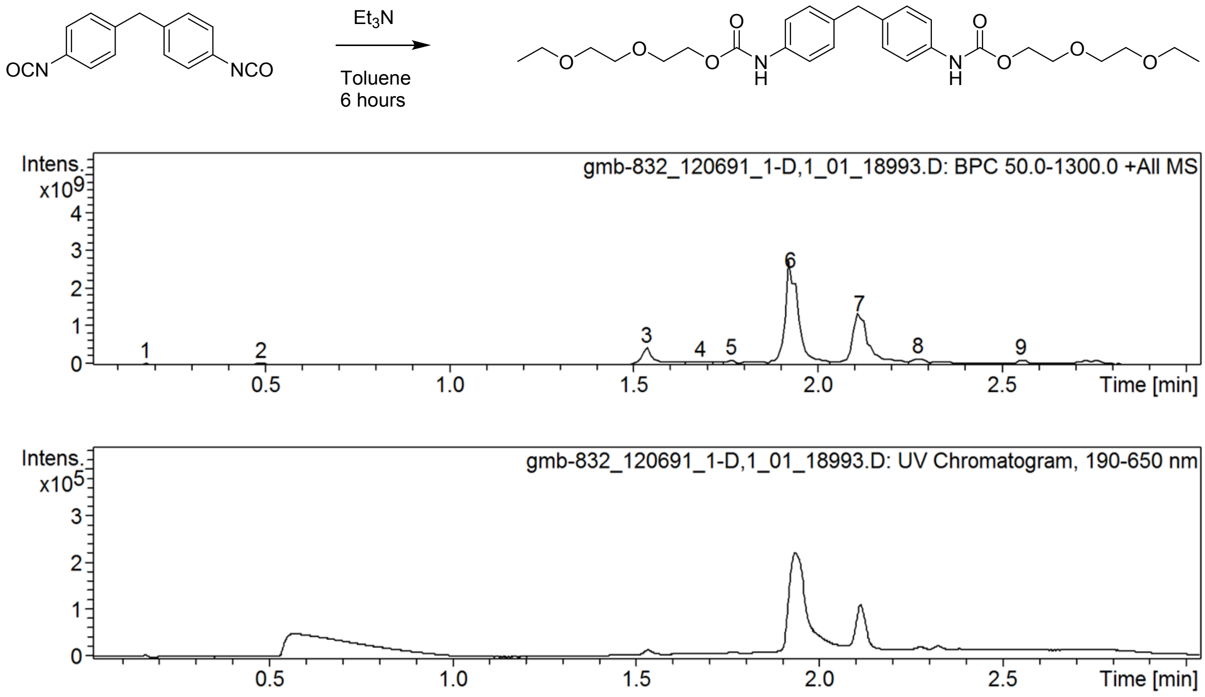

Fig. S19- Reaction of MDI **2** with diethyleneglycol monoethyl ether **8a** in toluene for 6 hours at 40 °C with triethylamine catalyst

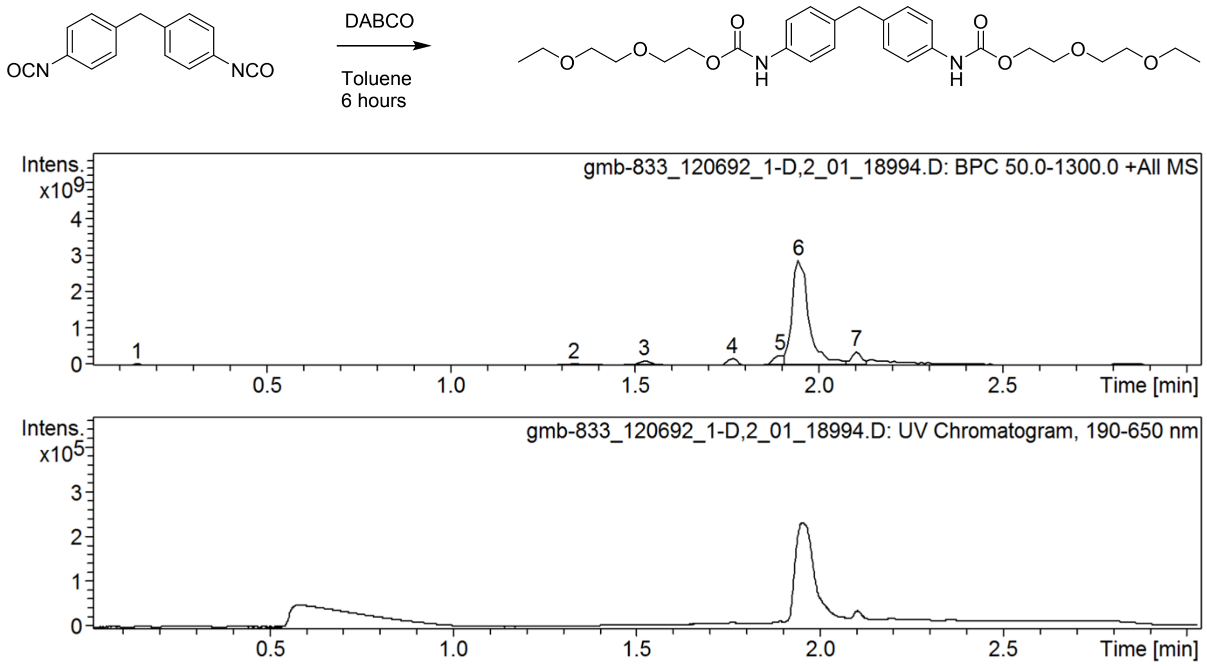

Fig. S20- Reaction of MDI **2** with diethyleneglycol monoethyl ether **8b** in toluene for 6 hours at 40 °C with DABCO catalyst

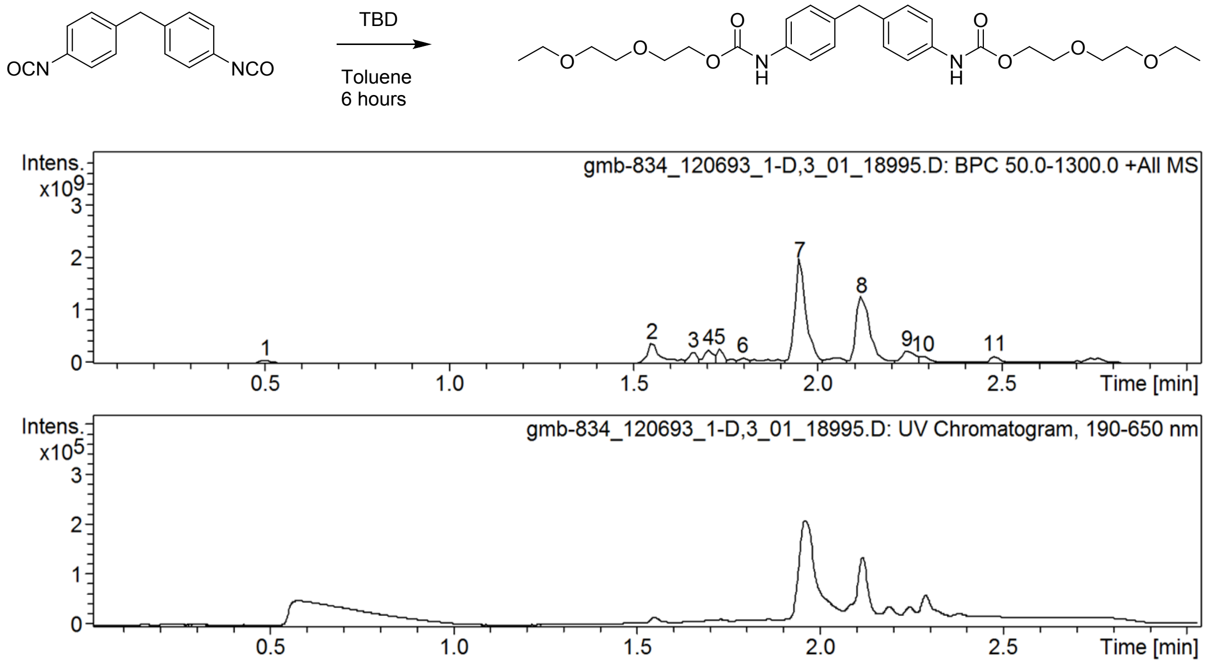

Fig. S21- Reaction of MDI **2** with diethyleneglycol monoethyl ether **8b** in toluene for 6 hours at 40 °C with TBD catalyst

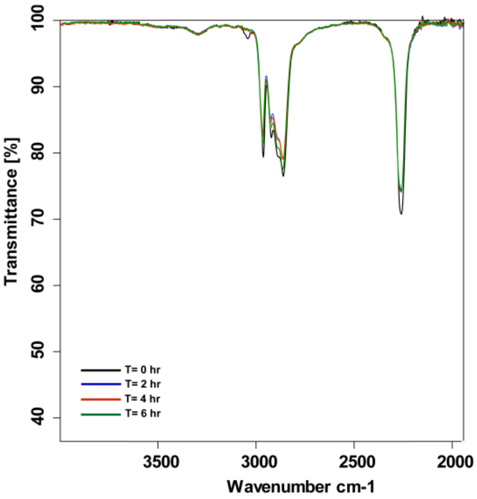

Fig. S22- Overlaid partial IR spectra of the reaction between MDI **2** and diol terminated PEG-PPG-PEG **1** in toluene at 40 °C with Et_3N catalysis for 6hr. In the reaction, –NCO functionality is retained, which is exhibited as a strong absorption at 2200cm^{-1}.

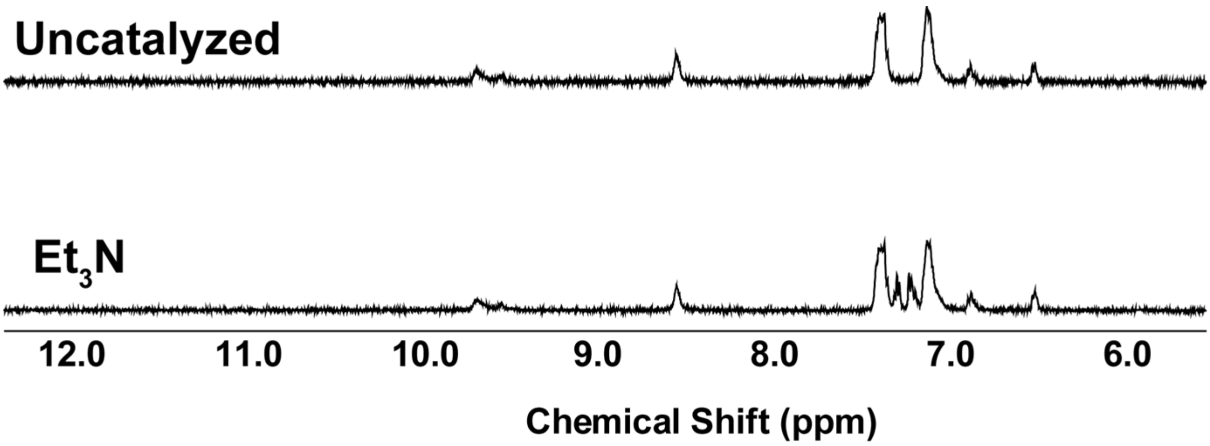

Fig. S23- the reaction between MDI **2** and diol terminated PEG-PPG-PEG **1** in toluene at room temperature. Partial crude NMR (DMSO-d_6, 500 MHz). Crude conversion was taken from integration of MDI protons from the desired product and starting material.

Catalyst	Reaction Progression/ %
**Uncatalyzed**	90
**Triethylamine**	88
**DABCO**	Insoluble
**TBD**	Insoluble

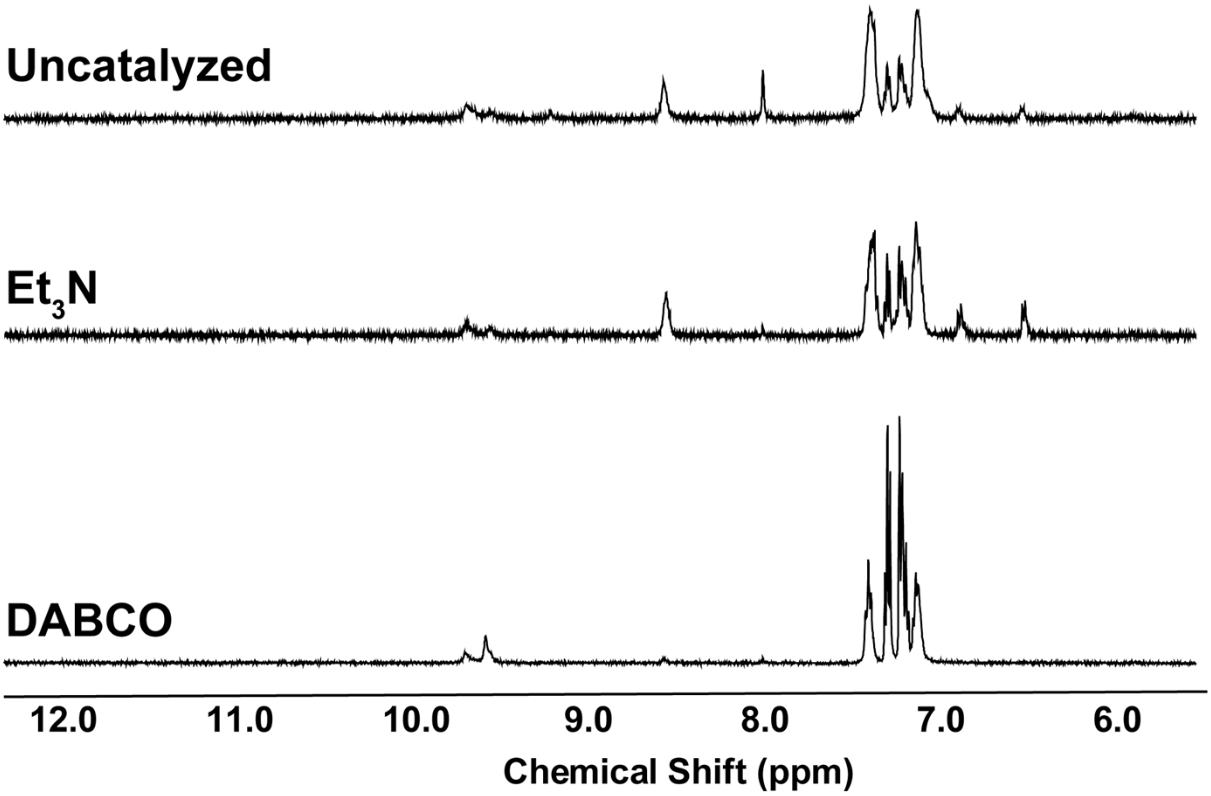

Fig. S24- reaction between MDI **2** and diol terminated PEG-PPG-PEG **1** in toluene at 40 °C. Partial crude NMR (DMSO-d_6, 500 MHz). Crude conversion was taken from integration of MDI protons from the desired product and starting material

Catalyst	Reaction Progression/ %
**Uncatalyzed**	95
**Triethylamine**	87
**DABCO**	100
**TBD**	Insoluble

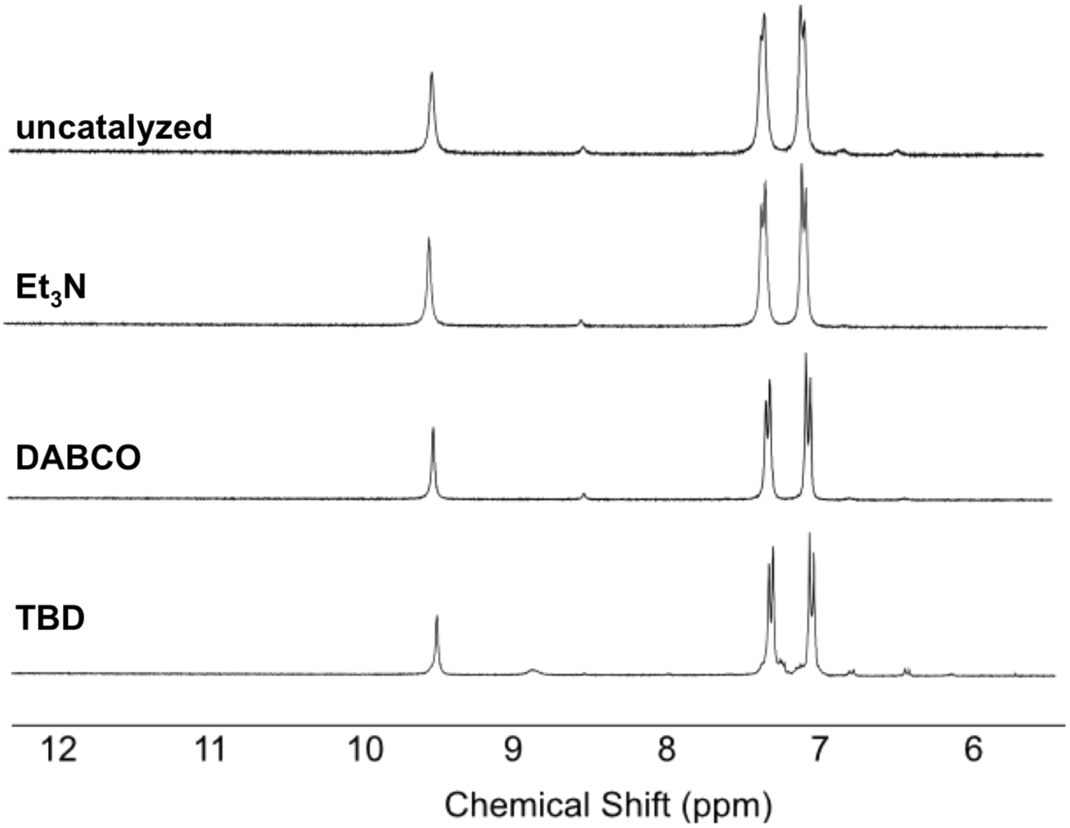

Fig. S25 - between MDI **2** and n-propanol **8a** in the ball mill at 20 Hz for 18 minutes. Partial crude NMR (DMSO-$d_{6,}$ 500 MHz); of the reaction. Crude conversion was taken from integration of MDI protons of the desired product (multiplet at 7.1ppm and 7.3ppm).

Catalyst	Conversion to Carbamate/ %
**Uncatalyzed**	93
**Triethylamine**	95
**DABCO**	93
**TBD**	80

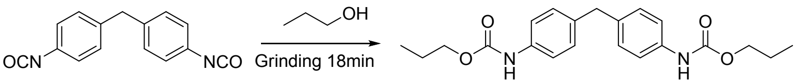

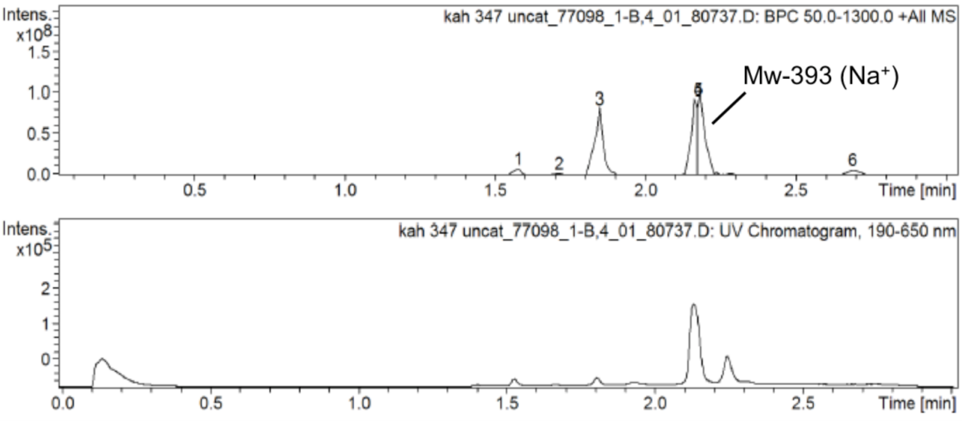

Fig. S26- Reaction of MDI **2** with 1-propanol **8a** in the ball mill for 18 minutes at 20 Hz with no catalyst

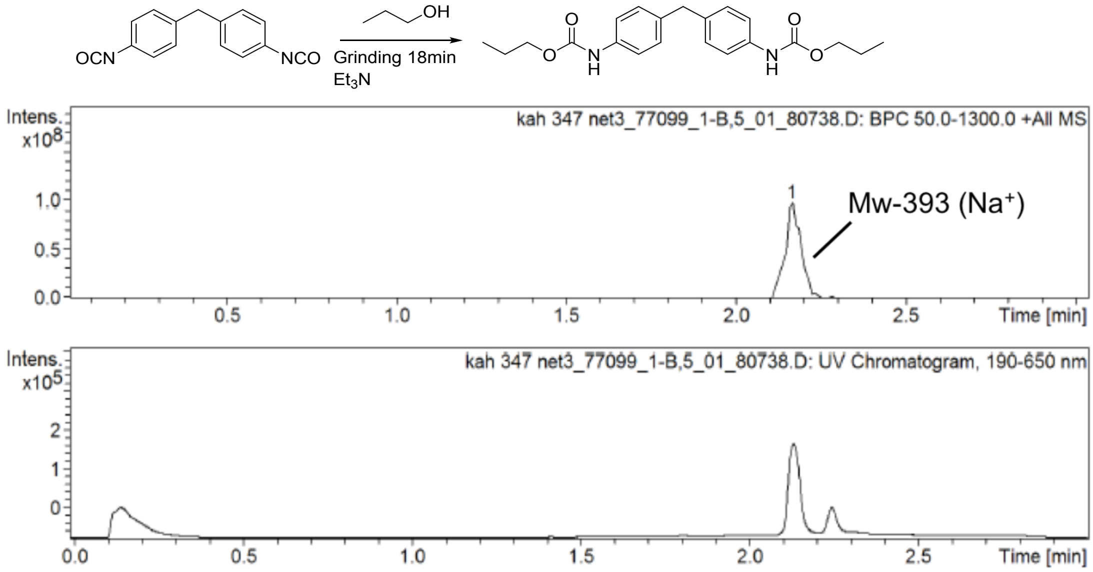

Fig. S27- Reaction of MDI **2** with 1-propanol **8a** in the ball mill for 18 minutes at 20 Hz with triethylamine catalyst

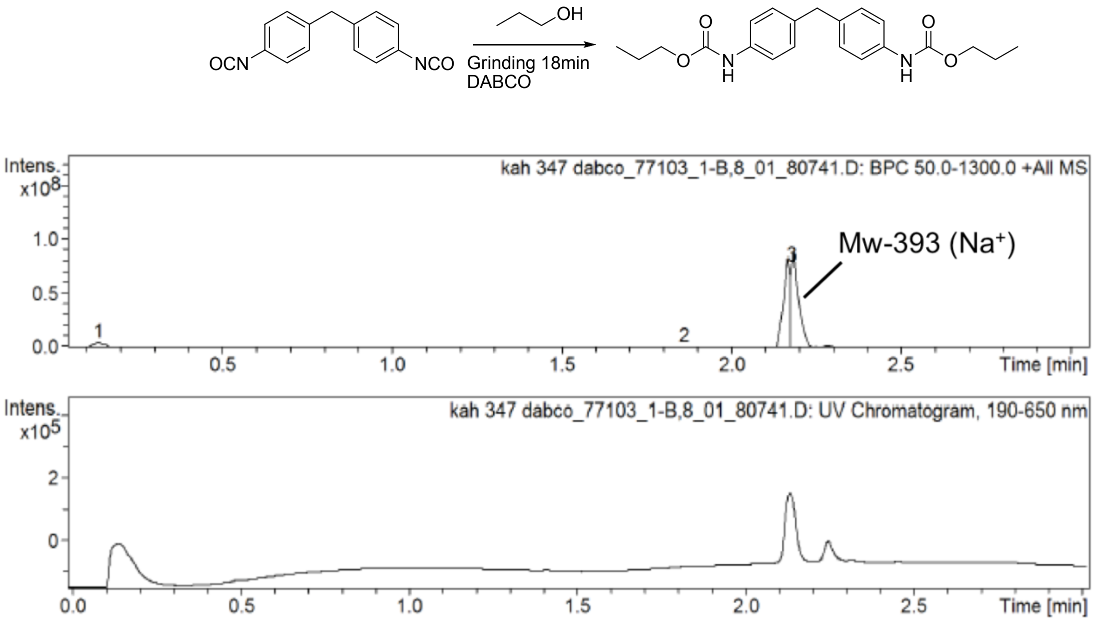

Fig. S28- Reaction of MDI **2** with 1-propanol **8a** in the ball mill for 18 minutes at 20 Hz with DABCO catalyst

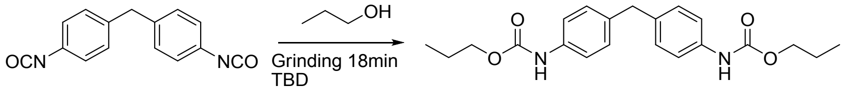

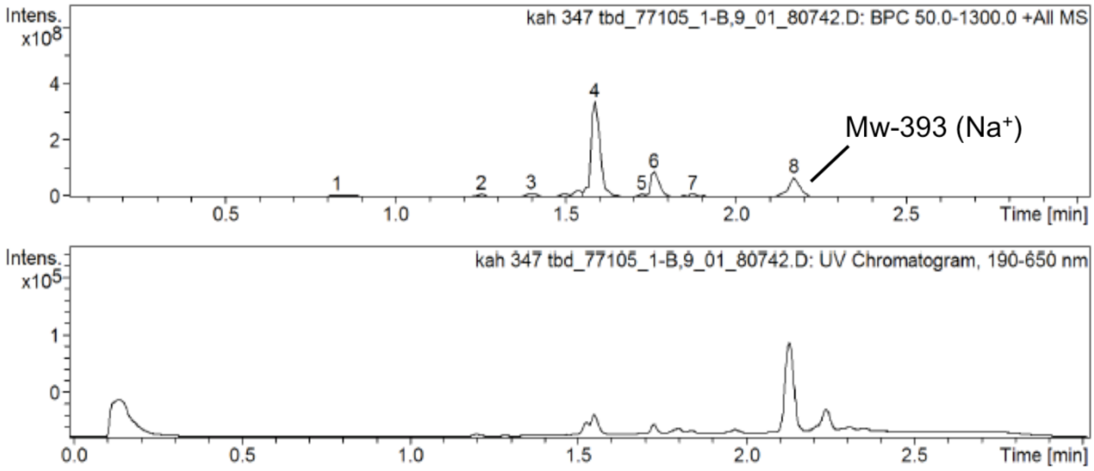

Fig. S29- Reaction of MDI **2** with 1-propanol **8a** in the ball mill for 18 at 20 Hz minutes with TBD catalyst

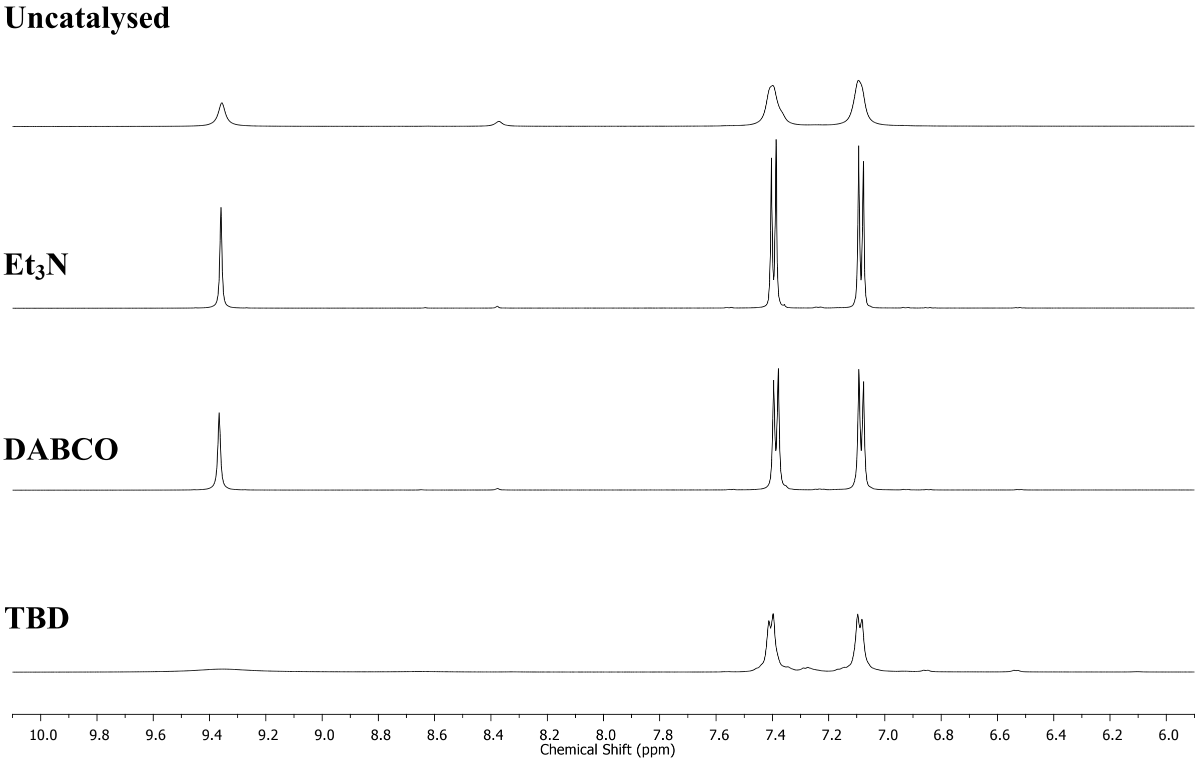

Fig. S30 - between MDI **2** and diethyleneglycol monoethyl ether **8b** in the ball mill at 20 Hz for 18 minutes. Partial crude NMR (DMSO-d_6, 500 MHz); of the reaction. Crude conversion was taken from integration of MDI protons of the desired product (multiplet at 7.1ppm and 7.3ppm).

Catalyst	Conversion to Carbamate/ %
**Uncatalyzed**	86
**Triethylamine**	98
**DABCO**	98
**TBD**	87

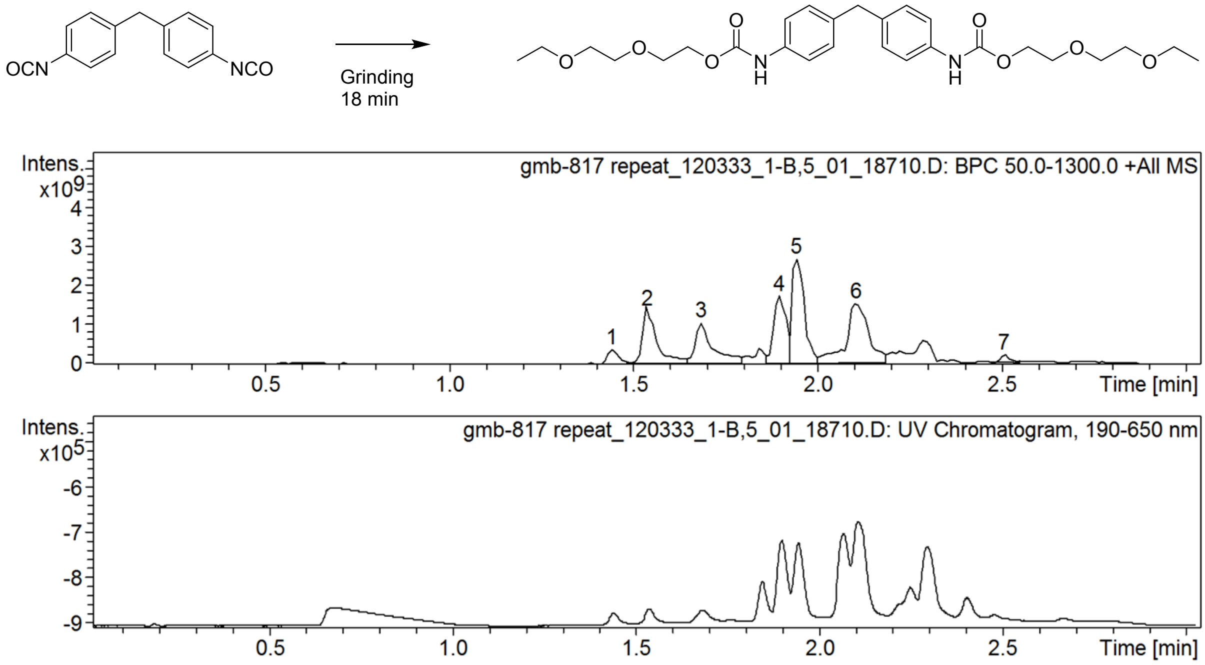

Fig. S31- Reaction of MDI **2** with diethyleneglycol monoethyl ether **8b** in the ball mill for 18 minutes at 20 Hz with no catalyst

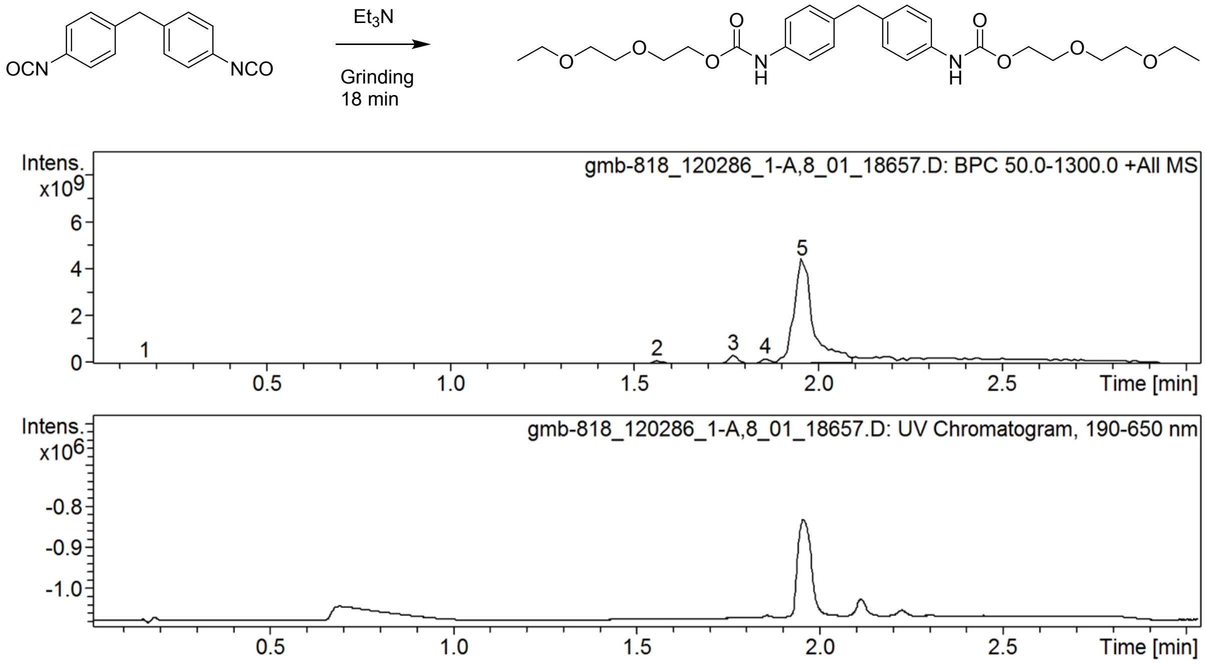

Fig. S32- Reaction of MDI **2** with diethyleneglycol monoethyl ether **8b** in the ball mill for 18 minutes at 20 Hz with triethylamine catalyst

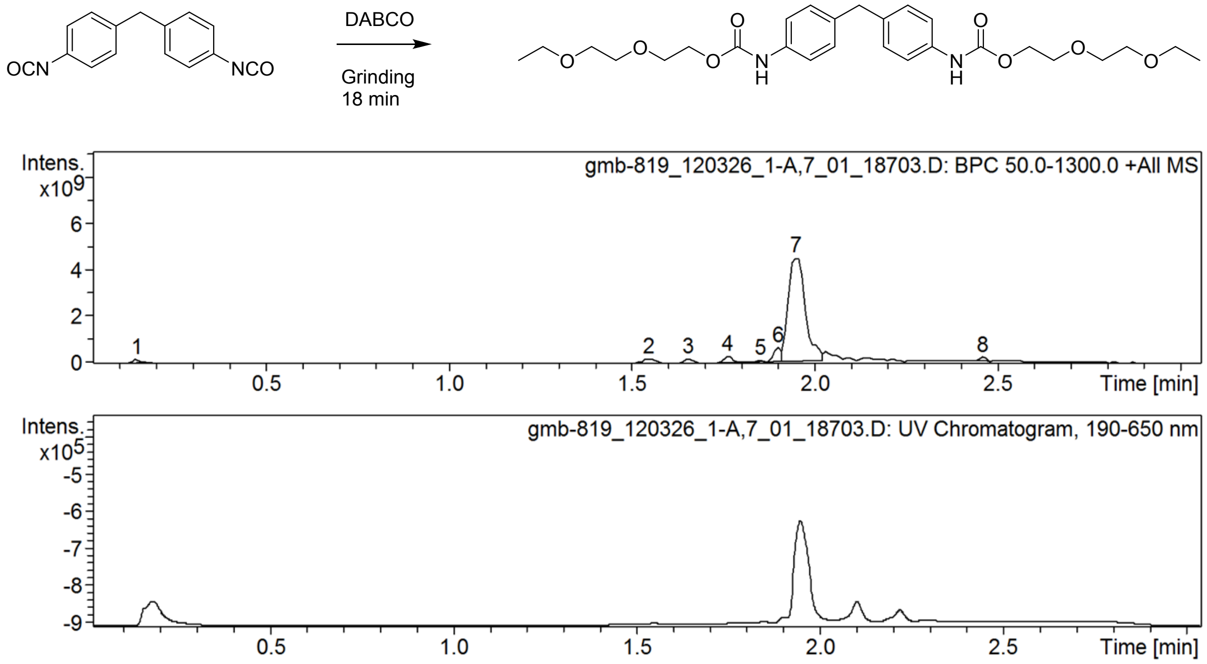

Fig. S33- Reaction of MDI **2** with diethyleneglycol monoethyl ether **8b** in the ball mill for 18 minutes at 20 Hz with DABCO catalyst

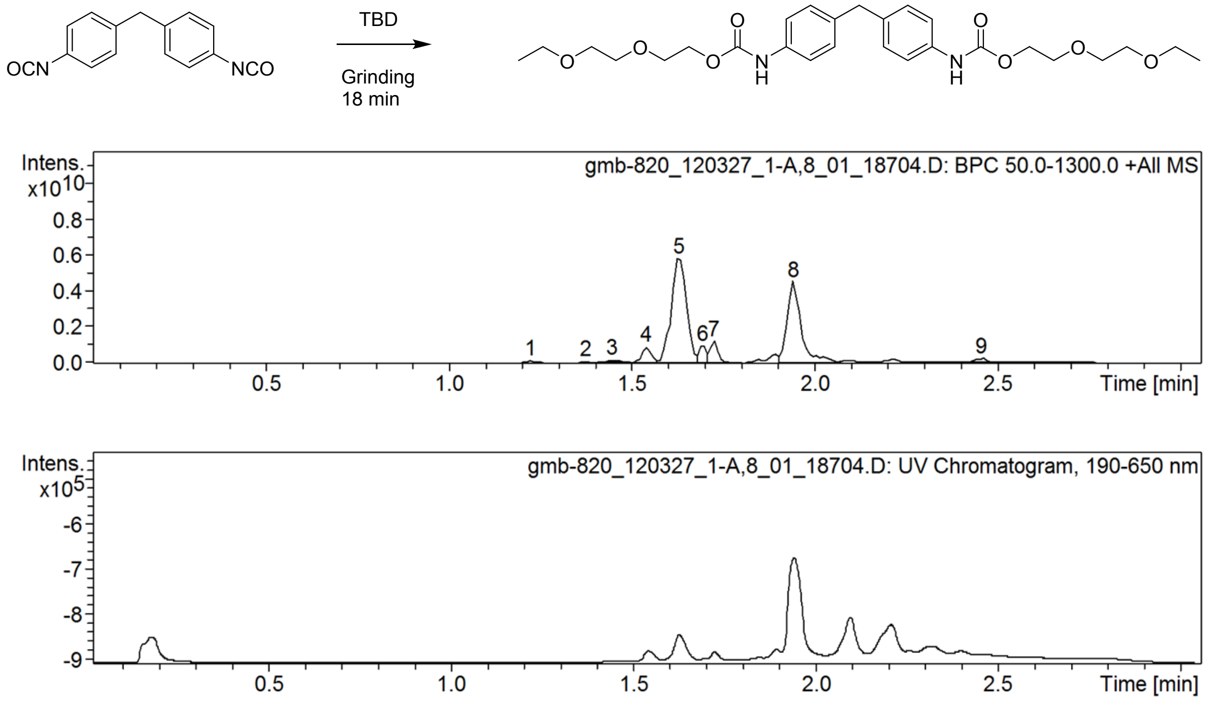

Fig. S34- Reaction of MDI **2** with diethyleneglycol monoethyl ether **8b** in the ball mill for 18 at 20 Hz minutes with TBD catalyst

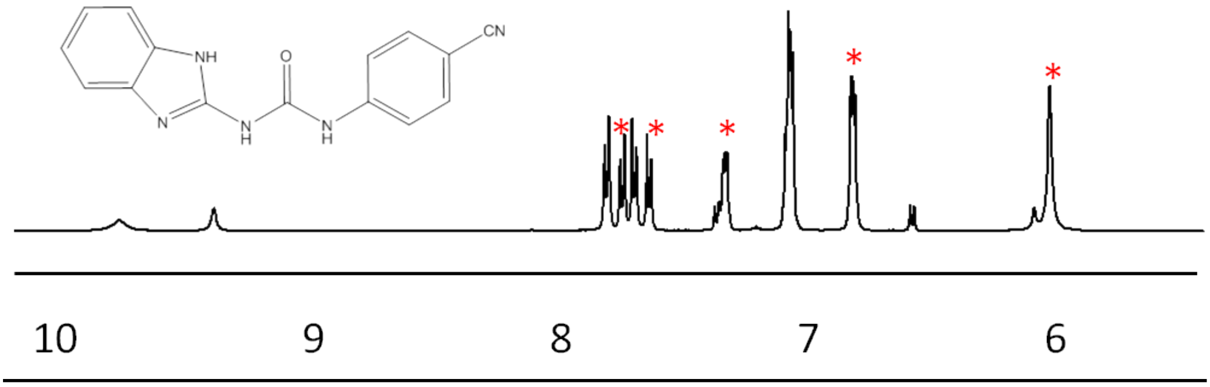

Fig. S35- reaction of p-cyano phenylisocyanate with 2-aminobenzimidazole in the ball mill for 10 minutes to give **11a**. Partial crude NMR (DMSO-$d_{6,}$ 500 MHz). Crude conversion was taken from integration of product protons (starred) against starting material.

Catalyst	Conversion to Carbamate/ %
**None**	46

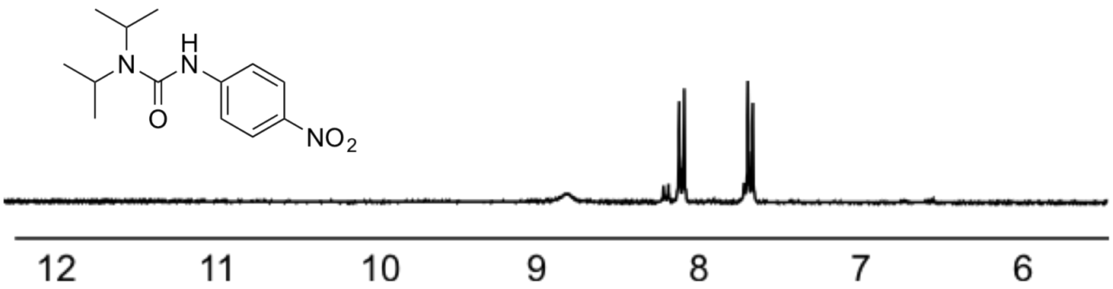

Fig. S36- reaction of p-nitro phenylisocyanate with diisopropylamine in the ball mill for 10 minutes to give **11b**. Partial crude NMR (DMSO-$d_{6,}$ 500 MHz).

Catalyst	Conversion to Carbamate/ %
**None**	99

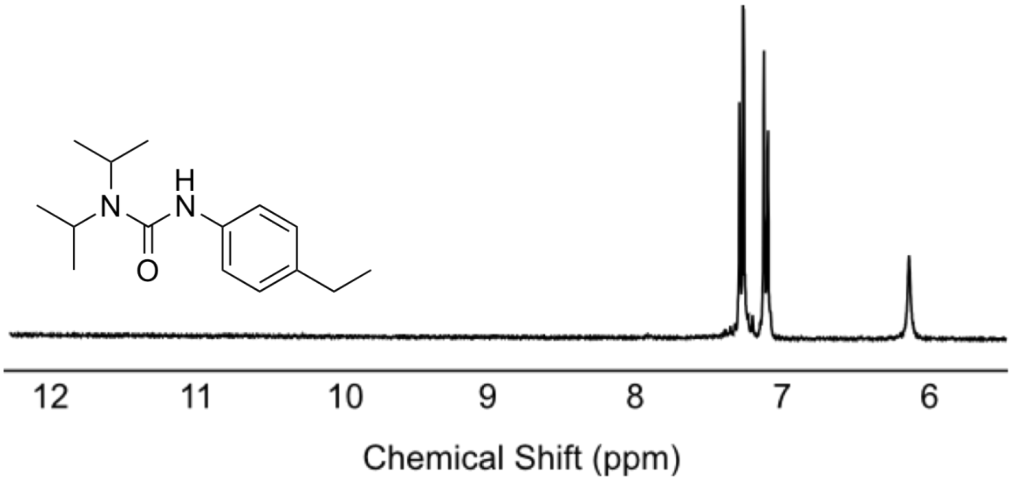

Fig. S37 Reaction of p-ethyl phenylisocyanate with diisopropylamine in the ball mill for 10 minutes to give **11c**. Partial crude NMR (DMSO-$d_{6,}$ 500 MHz)

Catalyst	Conversion to Carbamate/ %
**None**	100

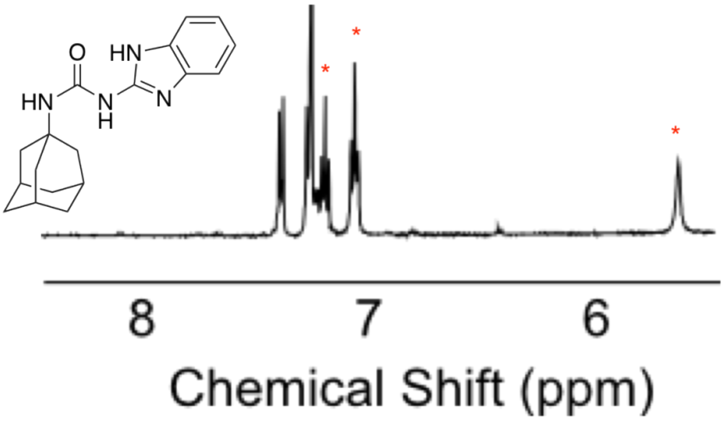

Fig. S38- Reaction of adamantyl isocyanate with 2-aminobenzimidazole in the ball mill for 10 minutes to give **11d**. Crude conversion was taken from integration of product protons (starred) against starting material (DMSO-$d_{6,}$ 500 MHz).

Catalyst	Conversion to Carbamate/ %
**None**	60

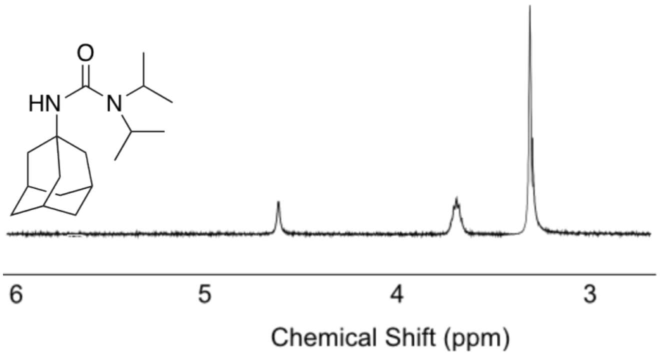

Fig. S39- Reaction of adamantyl isocyanate with diisopropylamine in the ball mill for 10 minutes to give **11e**. Partial crude NMR (DMSO-d_6, 500 MHz);

Catalyst	Conversion to Carbamate/ %
None	100

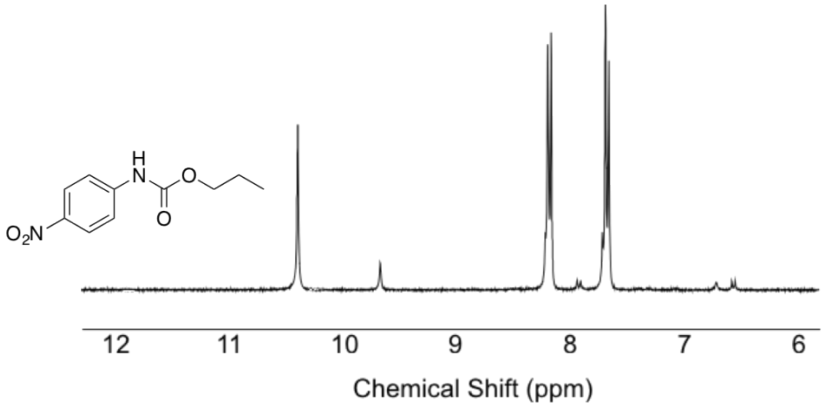

Fig. S40- Reaction of p-nitro phenylisocyanate with n-propanol in the ball mill for 10 minutes to give **12a**. Partial crude NMR (DMSO-d_6, 500 MHz)

Catalyst	Conversion to Carbamate/ %
None	79

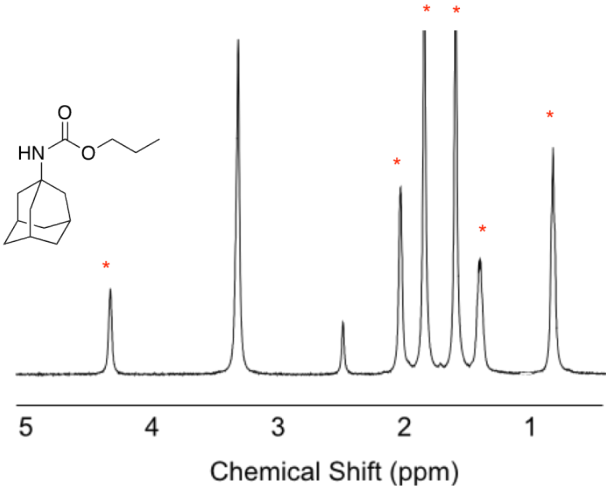

Fig. S41- Reaction of adamantyl isocyanate with n-propanol in the ball mill for 10 minutes to give **12b**. Partial crude NMR (DMSO-d_6, 500 MHz); Crude conversion was taken from integration of product protons (starred) against starting material.

Catalyst	Conversion to Carbamate/ %
**None**	100

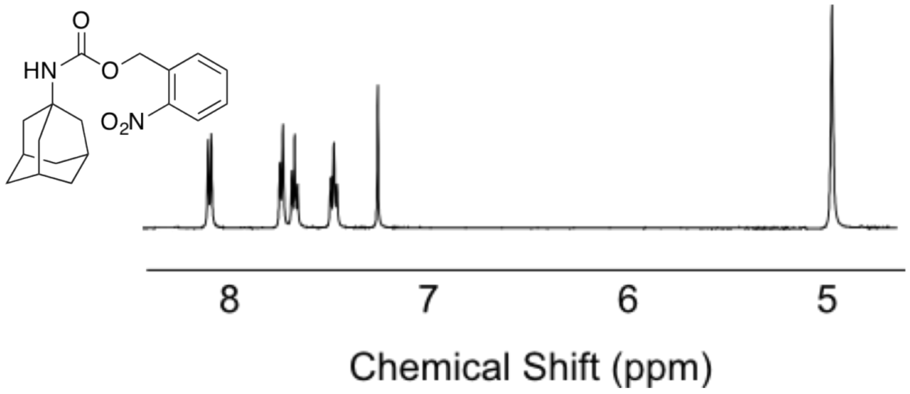

Fig. S42- Reaction of adamantyl isocyanate with O-nitro benzylalcohol in the ball mill for 10 minutes to give **12c**. Partial crude NMR ($CDCl_3$, 500 MHz).

Catalyst	Conversion to Carbamate/ %
**None**	100

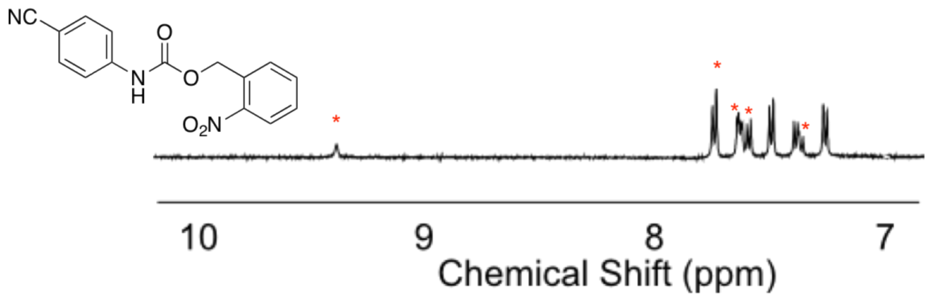

Fig. S43- Reaction of p-cyano phenylisocyanate with o-nitrobenzyl alcohol in the ball mill for 10 minutes to give **12d**. Partial crude NMR (DMSO-d_6, 500 MHz): Crude conversion was taken from integration of product protons (starred) against starting material.

Catalyst	Conversion to Carbamate/ %
**None**	50

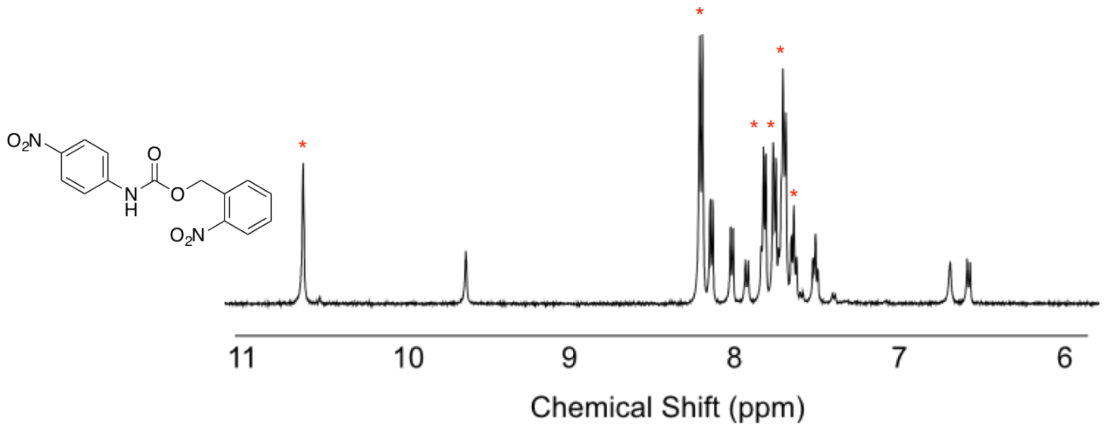

Fig. S44 Reaction of p-nitro phenylisocyanate with o-nitro benzylalcohol in the ball mill for 10 minutes to give **12e**. Partial crude NMR (DMSO-d_6, 500 MHz); Crude conversion was taken from integration of product protons (starred) against starting material.

Catalyst	Conversion to Carbamate/ %
**None**	75

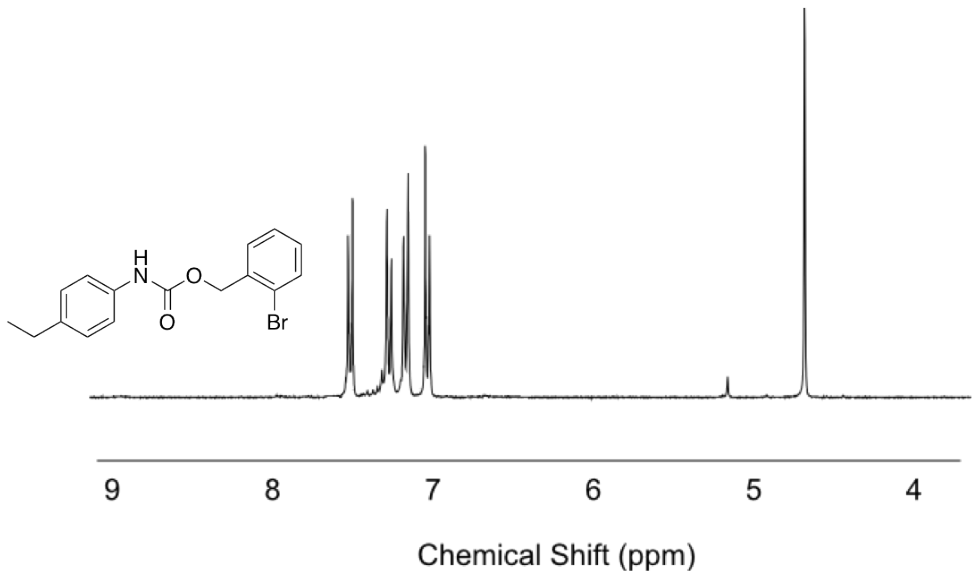

Fig. S45- Reaction of p-ethyl phenylisocyanate with o-bromo benzylalcohol in the ball mill for 10 minutes to give **12f**. Partial crude NMR ($CDCl_3$, 500 MHz);

Catalyst	Conversion to Carbamate/ %
**None**	55

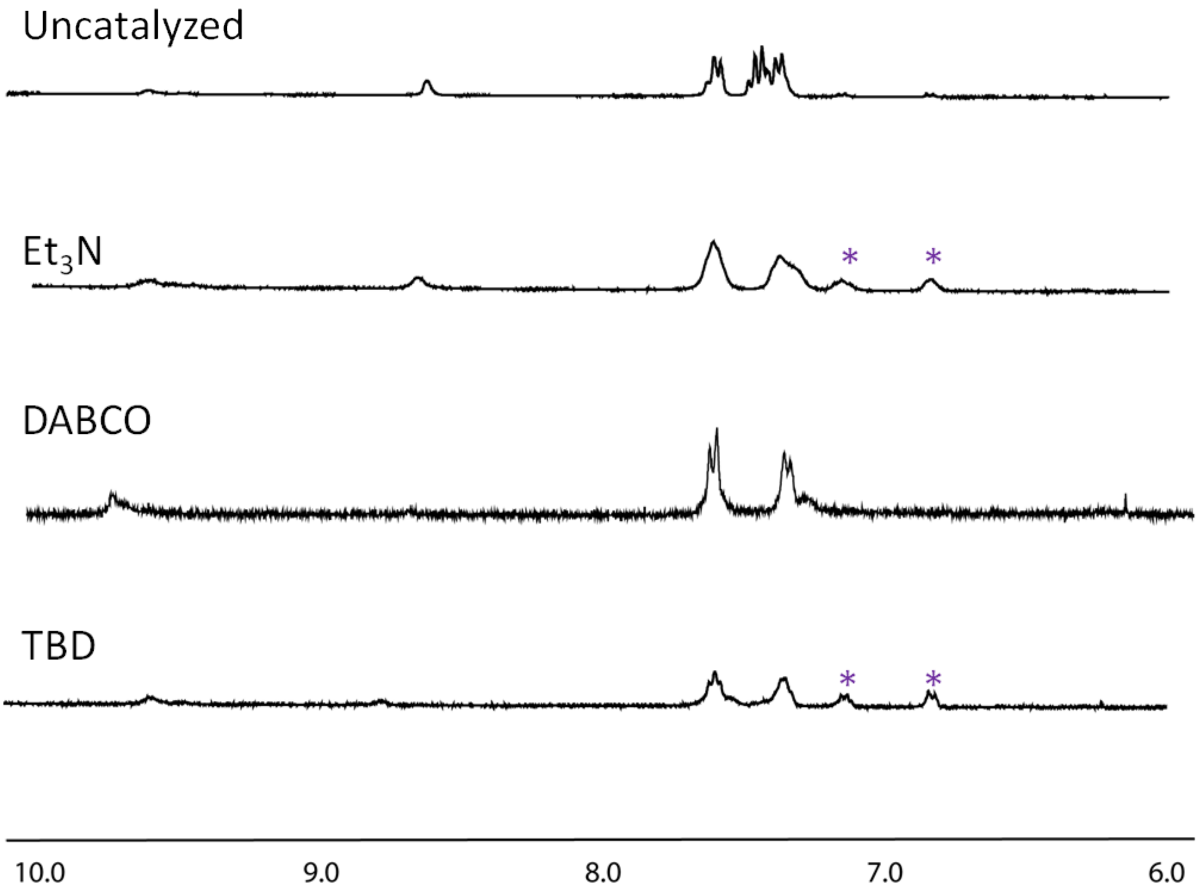

Fig. S46- Demonstrating catalyst effect on polyurethane **3a** formation in the ball mill for 30min. Partial crude NMR (DMSO-d_6, 500 MHz); Crude conversion was taken by integrating MDI protons in the desired product (multiplets at 7.1ppm and 7.3ppm) against unreacted material. * indicative of starting material

Catalyst	Conversion to Urethane/ %
**Uncatalyzed**	70
**Triethylamine**	78
**DABCO**	100
**TBD**	93

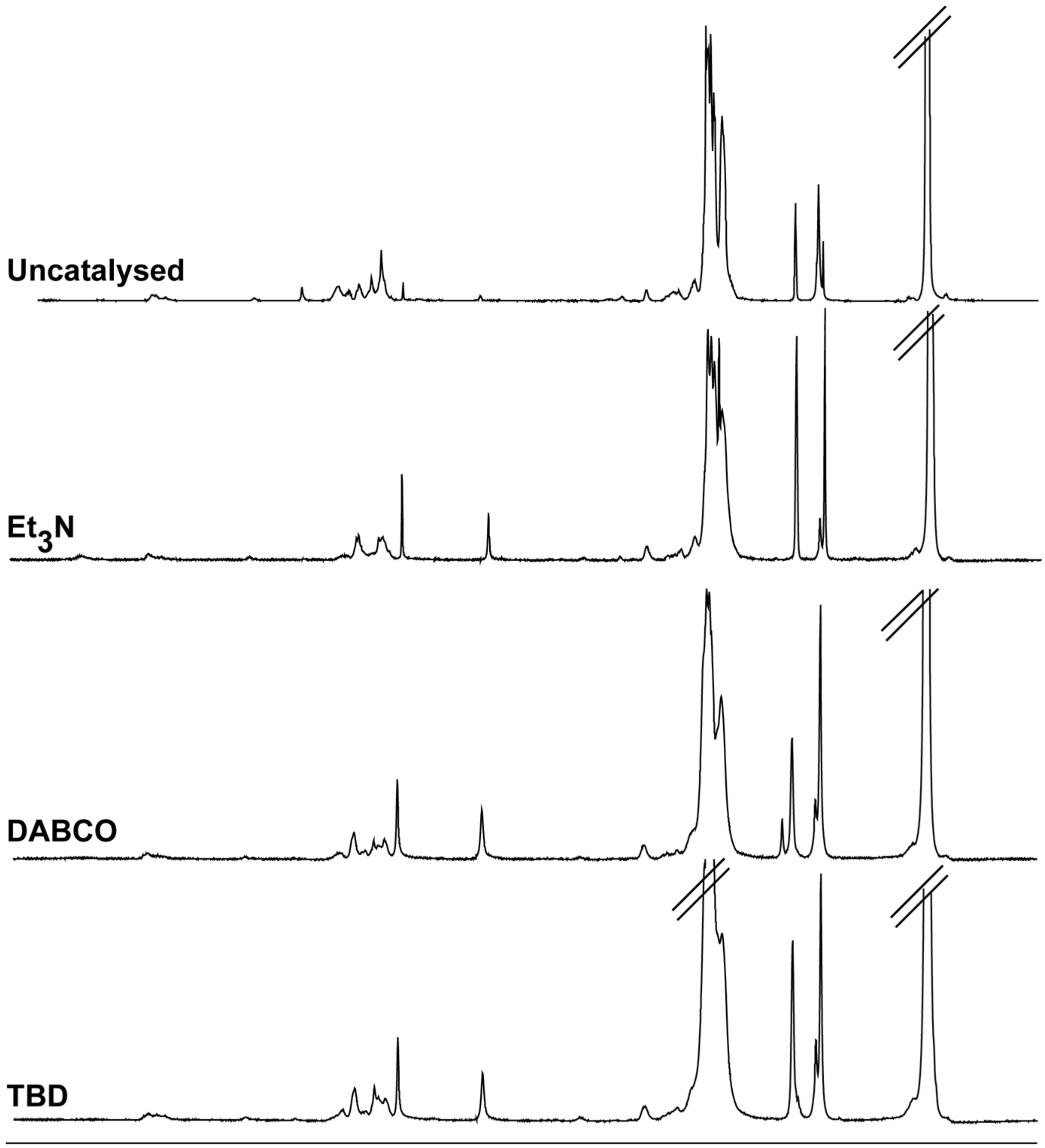

Fig. S47 Demonstrating catalyst effect on macromonomer, **5a** formation in the ball mill for 10min. Crude NMR (DMSO-d_6, 500 MHz);

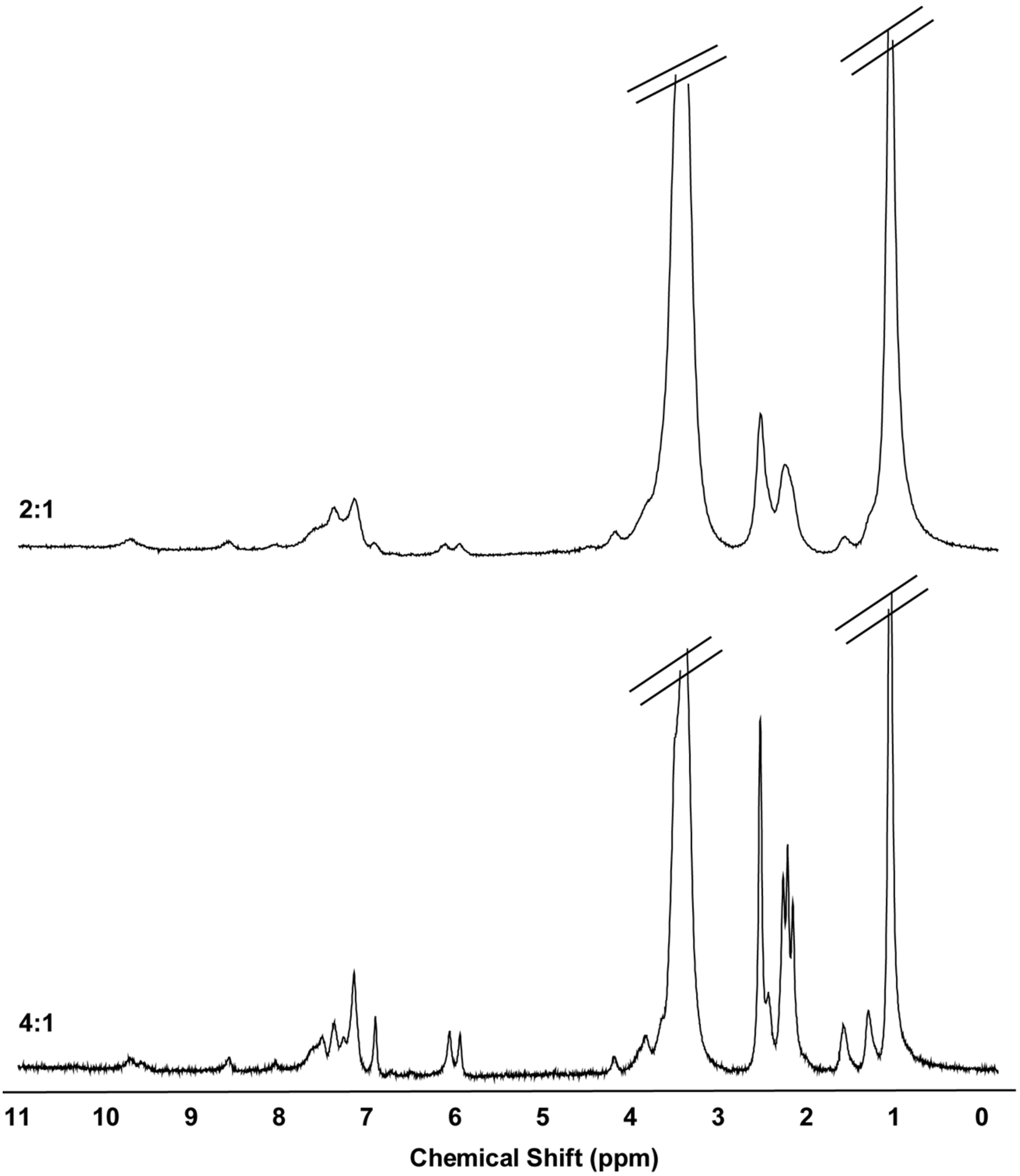

Fig. S48 Full spectrum of supramolecular polymers, **7a** and **7b,** formed in the ball mill. Crude NMR (DMSO-d_6, 500 MHz);

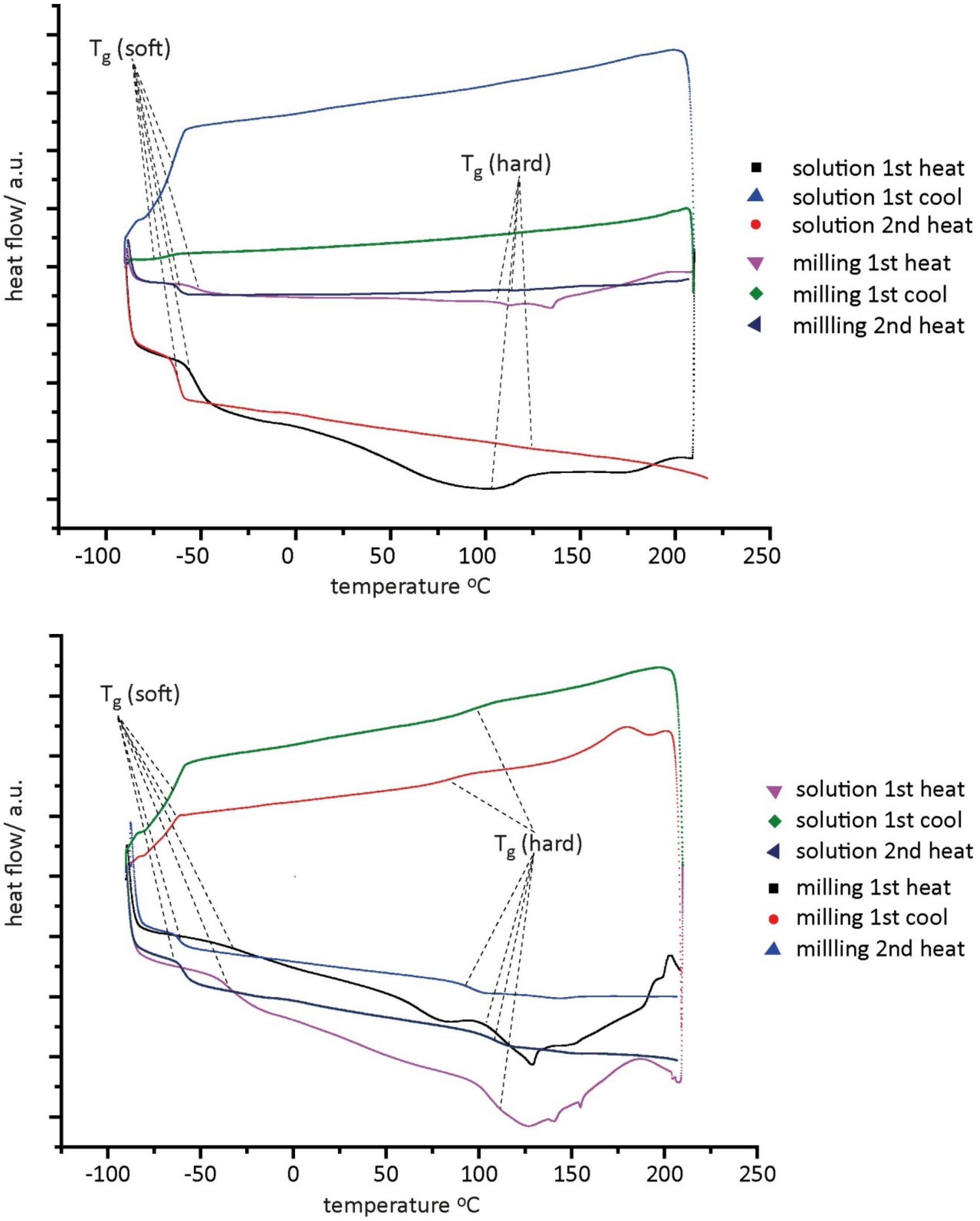

Fig. S49 Differential Scanning Calorimetry (DSC) scans of supramolecular polymers formed synthesized in a ball mall solvent-free using a 2:1 NCO to OH ratio **7a** (top) and 4:1 NCO to OH ratio **7b** (bottom). Scans were conducted between -90 °C and 210 °C, at a heating and cooling rate of 10 deg min^{-1} after N_2 purge. Comparison is made to supramolecular polymers, which are synthesized using standard solution methodology.

References

1. A. Gooch, C. Nedolisa, K. A. Houton, C. I. Lindsay, A. Saiani and A. J. Wilson, *Macromolecules*, 2012, **45**, 4723-4729.
2. A. Gooch, S. Barrett, J. Fisher, C. I. Lindsay and A. J. Wilson, *Org. Biomol. Chem.*, 2011, **9**, 5938-5940.
